# Comprehensive Biodegradation Analysis of Chemically Modified Poly(3-hydroxybutyrate) Materials with Different Crystal Structures

**DOI:** 10.1021/acs.biomac.3c00623

**Published:** 2023-10-11

**Authors:** Markéta Julinová, Dagmar Šašinková, Antonín Minařík, Martina Kaszonyiová, Alena Kalendová, Markéta Kadlečková, Ahmad Fayyazbakhsh, Marek Koutný

**Affiliations:** † Department of Environmental Protection Engineering, Faculty of Technology, Tomas Bata University in Zlín, Nad Ovčírnou 3685, 760 01, Zlín, Czech Republic; ‡ Department of Physics and Material Engineering, Faculty of Technology, Tomas Bata University in Zlín, Vavrečkova 5669, 760 01, Zlin, Czech Republic; § Department of Polymer Engineering, Faculty of Technology, Tomas Bata University in Zlín, Vavrečkova 5669, 760 01, Zlín, Czech Republic

## Abstract

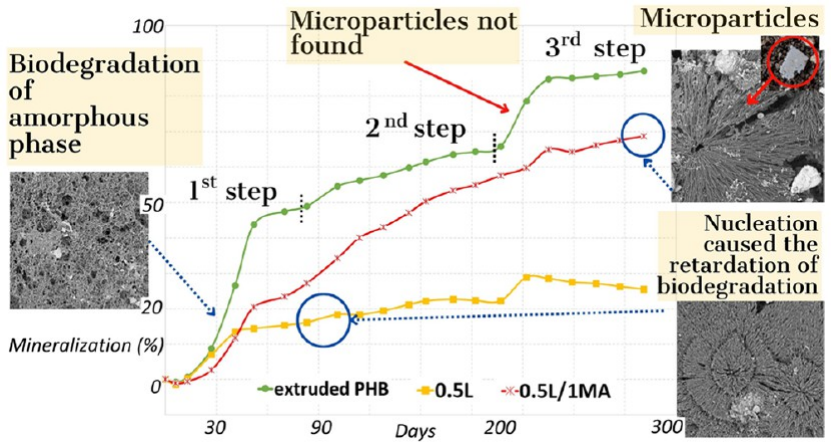

This work presents a comprehensive analysis of the biodegradation of polyhydroxybutyrate (PHB) and chemically modified PHB with different chemical and crystal structures in a soil environment. A polymer modification reaction was performed during preparation of the chemically modified PHB films, utilizing 2,5-dimethyl-2,5-di(*tert*-butylperoxy)-hexane as a free-radical initiator and maleic anhydride. Films of neat PHB and chemically modified PHB were prepared by extrusion and thermocompression. The biological agent employed was natural mixed microflora in the form of garden soil. The course and extent of biodegradation of the films was investigated by applying various techniques, as follows: a respirometry test to determine the production of carbon dioxide through microbial degradation; scanning electron microscopy (SEM); optical microscopy; fluorescence microscopy; differential scanning calorimetry (DSC); and X-ray diffraction (XRD). Next-generation sequencing was carried out to study the microbial community involved in biodegradation of the films. Findings from the respirometry test indicated that biodegradation of the extruded and chemically modified PHB followed a multistage (2–3) course, which varied according to the spatial distribution of amorphous and crystalline regions and their spherulitic morphology. SEM and polarized optical microscopy (POM) confirmed that the rate of biodegradation depended on the availability of the amorphous phase in the interspherulitic region and the width of the interlamellar region in the first stage, while dependence on the size of spherulites and thickness of spherulitic lamellae was evident in the second stage. X-ray diffraction revealed that orthorhombic α-form crystals with helical chain conformation degraded concurrently with β-form crystals with planar zigzag conformation. The nucleation of PHB crystals after 90 days of biodegradation was identified by DSC and POM, a phenomenon which impeded biodegradation. Fluorescence microscopy evidenced that the crystal structure of PHB affected the physiological behavior of soil microorganisms in contact with the surfaces of the films.

## 1 Introduction

It is known that the biodegradability of an aliphatic polyester (PHA), produced by biosynthesis and chemosynthesis, is strongly connected with aspects of polymer morphology, e.g., crystallinity, molecular orientation, chain packing, and the crystal surface, in addition to chemical structure.^1^ Despite the history of such investigations over the last 30 years,^2^ the problem of microbial degradation in PHA is too far from a final resolution.

Polyhydroxybutyrate (PHB), discovered by French scientist Maurice Lemoigne in 1926, is the simplest, most common polyester in the PHA family. It has been very widely studied, and numerous references in the literature mention the plastic in connection with sustainability. PHB is a thermoplastic, one that is nontoxic, optically active and yellowish in hue when in a pure state. A semicrystalline polymer, PHB exhibits an elevated melting point and high crystallinity (up to 80%). Fabrication by thermal processing instigates the creation of crystalline domains in the form of spherulites of various sizes. The foremost property of PHB relates to ecology, though, as it can be completely degraded to CO_2_ and H_2_O by the action of microorganisms. Several studies have been published on the degradation of PHB and its composites in composting and natural ecosystems, such as soil and sediment or fresh/salt water.^1,3,4^ The microorganisms that degrade PHB belong to the Gram-positive and Gram-negative bacteria *Streptomyus* and fungi. PHB can be degraded by 39 bacterial strains of class *Firmicutes* and *Proteobacteria*, for example, *Pseudomonas lemoignei*, *Streptomyces* sp. SNG9, *Alcaligenes faecalis*, *Pseudomonas stutzeri*, and *Fusarium solani*.^5^

Data are available on the morphology and enzymatic degradation of PHB, though not particularly recent.^2,6−10^ These authors uniformly state that extracellular poly(hydroxybutyrate) depolymerases isolated from various environments degrade PHB materials first in their amorphous regions and subsequently in crystalline regions. Kumagai et al.,^6^ Tomasi et al.,^7^ and Abe et al.^8^ also describe the significant influence exerted by crystal size and related morphology (see below).

The effect of crystallinity and spherulite size on the enzymatic degradation of melt-crystallized PHB films was investigated by Kumagai et al.,^6^ under conditions of 37 °C and pH 7.4 in aqueous solutions of extracellular PHB depolymerase isolated from *Alcaligenes faecalis* T1; They found that the rate of enzymatic degradation of the PHB films diminished in parallel with an increase in crystallinity, yet the size of the spherulites had a negligible effect. They proposed that the PHB depolymerase initially hydrolyzed the PHB chains in the amorphous phase on the surfaces of films, and then eroded such chains in the crystalline state. In contrast, Tomasi et al.^7^ reported that the rate of enzymatic degradation at 37 °C for melt-crystallized PHB films decreased alongside a rise in the mean sizes of crystals; the films in question had been obtained by compression molding two types of PHB depolymerase isolated from *Pseudomonas lemoignei*.

Abe et al.^8^ studied the enzymatic degradation of melt-crystallized films synthesized from copolymers of (*R*)-3-hydroxybutyric acid and various hydroxyalkanoic acids at 37 °C in an aqueous solution (pH 7.4) of PHB depolymerase isolated from *Alcaligenes faecalis*. They reported that, following enzymatic degradation, smooth and rough planes coexisted in the radial direction of spherulites on the surfaces of the melt-crystallized films. This indicated that PHB depolymerase predominantly hydrolyzed polymer chains on the edges of crystalline lamellar stacks. The rate of enzymatic erosion affecting the crystalline region in the polyester films dropped with an increase in lamellar thickness.

Probably the most relevant data for the research conducted herein was reported by Iwata et al.^11^ The team described the adsorption of two extracellular PHB depolymerases isolated from *Comamonas acidovorans* YM1609 and *Alcaligenes faecalis* T1 on PHB monocrystals by immunogold staining in a work.^11^ Homogeneous distribution of enzymatic molecules on the chain-folding surfaces of the crystals was demonstrated; however, enzymatic degradation of the latter progressed from the edges to their long axes (not crystal surfaces), forming minute crystalline fragments, despite the occurrence of homogeneous adsorption of the molecules on such surfaces. The results published by Iwata et al.^11^ suggested that an enzymatic molecule was incapable of degrading an ester unit in the chain-folding portion of the molecular chain on the crystal surface by steric hindrance. In later research, Iwata et al.^10^ studied the enzymatic degradation of PHB fibers that had a core–sheath structure with two molecular conformations: α-form (2/1 helix) and β-form (planar zigzag). The core region consisted of both of these, with only the α-form in the sheath region. An extracellular poly(hydroxybutyrate) depolymerase purified from *Ralstonia pickettii* T1 was applied to instigate enzymatic degradation of the fibers. The sequence of such degradation progressed, thus, it commenced with amorphous chains between α-form lamellar crystals in the sheath region, moving on to β-form molecular chains in the core region, and ended with α-form lamellar crystals throughout the fiber. Their conclusion was that the rate of enzymatic erosion of the β-form was more rapid than the α-form, indicating that the rate of enzymatic degradation could be controlled by molecular conformation, even though the chemical structures of the tested fibers were identical.

From the literary research conducted, it is clear that key aspects of morphology, such as crystallinity, molecular orientation, chain packing, crystal surface, and size, affect the rate of PHB enzymatic degradation of materials identical in the chemical structure. The course and rapidity of biodegradation in a natural ecosystem, however, can differ significantly from enzymatic degradation in laboratory experiments.

Therefore, in order to extend the applicability of PHB and associated composites, it is necessary to understand how different morphologies that arise through crystallization behavior influence the biodegradation of PHB-based products in a real-world environment with immense diversity in microorganisms.

As previously mentioned, several studies exist on the enzymatic degradation of PHB in relation to morphology or the environmental biodegradation of PHB-based materials in connection with chemical structure. As far as the authors are aware, though, no detailed work has been published on the mutual effects of chemical structure and morphology, i.e., crystallinity, chain packing, and crystal surface, on the biodegradation of PHB by a natural, diverse microbial community. Given that it is now possible to analyze the composition and dynamics of the microbial communities into great detail, thanks to modern cultivation-independent methods based on the next-generation sequencing of the nucleic acids isolated from the samples and availability of these techniques in our laboratory, we decided to use this methodology to study differences of the polymer degrading microbial communities between different samples. As discussed above, the changes in morphology and crystallinity indeed are reflected in the rate of the biodegradation, and there is a hypothesis that they could also alter the composition of the microbial community.

As a consequence, polymer films were prepared that underwent biodegradation, with the investigation concentrating on the roles of chemical structure and morphology. According to Przybysz–Romatowska et al.^12^ and Chen et al.,^13^ chemical modification by the action of free-radical initiators and reactive monomers is a means of fabricating PHB materials that differ in chemical structure and crystal morphology. Applying this methodology herein led to the creation of samples of chemically modified PHB films through such a polymer modification reaction, utilizing 2,5-dimethyl-2,5-di(*tert*-butylperoxy)hexane (L) as the free-radical initiator^14^ and maleic anhydride (MA) as the reactive monomer.^13^ The course, rate, and level of biodegradation were analyzed by a respirometric test to gauge the production of carbon dioxide. Differential scanning calorimetry (DSC) and X-ray diffraction analysis (XRD) were carried out to discern changes in crystallinity instigated by biodegradation, while morphology was characterized by polarized optical microscopy (POM) and scanning electron microscopy (SEM). Alteration in the quality and quantity of microbiocenoses during biodegradation was also assessed by studying the surfaces of the materials by fluorescence microscopy and subsequent sequencing of the microbial communities present during the experiment.

## 2 Materials and Methods

### 2.1 Materials and Chemicals

The following were utilized in the preparation of the polymer films: poly-3-hydroxybutyrate (technical grade and additive-free, *M*_n_ 87910 g·mol^–1^, *M*_w_ 437900 g·mol^–1^, *M*_*z*_ 1350000 g·mol^–1^, the dispersity index of 4.98 as revealed by GPC), biosynthesized from *Cupriavidus necator* with d-glucose as a medium (obtained from Tianan Biologic Materials Co., Ltd., China); the aforementioned 2,5-dimethyl-2,5-di(*tert*-butylperoxy)hexane (Luperox101) and maleic anhydride were supplied by Sigma-Aldrich.

The other chemicals employed were of analytical purity and sourced from PLIVA Lachema (Brno, Czech Republic).

### 2.2 Fabrication of the Materials

PHB and chemically modified PHB films were prepared by extrusion on a two screw extruder Labtech Engineering company, Ltd., at a temperature of 185 °C and 50 rpm under a nitrogen atmosphere. Films of melt-processed samples were obtained by thermocompression on a hydraulic press at 185 °C and 15 MPa, for a period of 5 min. Table 1 presents data on the samples and the basic characterization of them.

**Table 1:** Content of the Free-Radical Initiator 2,5-Dimethyl-2,5-di-(*tert*-butylperoxy)hexane (L) and Maleic Anhydride (MA) in the Samples in Wt (%) and Basic Characterization of the Films^a^

sample code	Luperox 101	maleic anhydride	TC^b^ (%)	thickness (μm)	gel fraction (%)
extruded PHB			56.12 ± 0.01	203.0 ± 2.0	4.35 ± 0.02
0.5L	0.5		56.09 ± 0.01	160.5 ± 16.0	10.49 ± 1.19
0.5L/0.5MA	0.5	0.5	55.96 ± 0.02	154.8 ± 14.0	16.74 ± 0.55
0.5L/1MA	0.5	1	55.87 ± 0.03	151.5 ± 9.0	12.39 ± 0.91
0.5L/1.5MA	0.5	1.5	55.91 ± 0.03	111.3 ± 14.9	3.23 ± 2.67
0.5L/2MA	0.5	2	55.71 ± 0.03	99.0 ± 0.8	2.93 ± 0.64
1L	1		56.08 ± 0.02	129.8 ± 20.0	16.67 ± 0.67
1L/0.5MA	1	0.5	55.98 ± 0.02	139.8 ± 9.0	11.94 ± 0.19
1L/1MA	1	1	55.97 ± 0.04	132.0 ± 3.0	11.35 ± 2.82
1L/1.5MA	1	1.5	55.74 ± 0.01	136.0 ± 6.0	3.01 ± 2.32
1L/2MA	1	2	55.75 ± 0.04	58.4 ± 16.6	5.47 ± 2.08

a *n* = 10, average ± standard deviation.

b TC: total carbon (Automatic Elemental Analyzer, Thermo Fisher Scientific Inc.).

### 2.3 Determination of the Gel Fraction

The gel content of the extruded PHB and chemically modified PHB films was determined according to a modified procedure by Dong et al.,^15^ involving the measurement by solvent extraction (in refluxing chloroform, 160 °C, 4 h), so as to determine the extent of modification caused by the presence of L and MA. The extract was passed 4 h later through a nylon filter (mesh 45 μm) to collect insoluble fractions, which were dried at 50 °C to constant weight, and the amount of the gel fraction was determined gravimetrically according to eq 1.
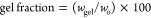
1where *w*_o_ is the original weight of the dry samples and *w*_gel_ is the weight of the dry gel fraction.

### 2.4 Biodegradation in Soil

Biologically active soil from a forest was sieved to remove any coarse matter, its moisture measuring ∼54%. The value for soil exchange capacity (pH_KCl_) was determined as 6.9, while the content of total carbon in the solid phase was 16.9 ± 1.36% (Automatic Elemental Analyzer, Thermo Fisher Scientific).

#### Respirometric Test

In accordance with a work by Šera et al.,^16^ a modified respirometric test with adherence to ISO 17556^17^ was conducted. Biometric flasks of size 500 mL with septa in the cap were used, and into each one was added 15 g of soil, 50 mg of polymer samples, perlite, a heat-expanded aluminosilicate (5 g), and mineral medium (10 mL). Head space gas was sampled through the septum with a gastight syringe and then conducted through a capillary tube into a gas analyzer (UAG, Stanford Instruments, U.S.A.) to determine the concentration of CO_2_. The bottles were stored at 25 °C, and measurement took place once a week from the commencement of the test, followed by once every 14 days. Triplicates of each sample were prepared, i.e., 3 parallel flasks, in conjunction with 4 blanks.^16^

The basic criterion in relation to CO_2_ and biodegradability concerned the ratio of how much gas was actually produced during microbial breakdown compared to a theoretical quantity (ThCO_2_), as given by the balance of carbon present in the sample (Table 1) expressed as *D*_CO_2__ (%), as in eqs 2 and 3 below:
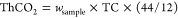
2where ThCO_2_ is the theoretical production of CO_2_ from total substrate breakdown (in mg), as determined by the balance of organically bound carbon in the tested material (TC in percent), *w*_sample_ represents the weight of the tested sample, 44 denotes the molecular weight of CO_2_ and 12 equals the molecular weight of carbon.
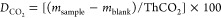
3where *m*_sample_ is the quantity of CO_2_ produced during breakdown of the tested films (mg), and *m*_blank_ stands for the quantity of CO_2_ produced during endogenous respiration of the microorganisms (mg).

#### Soil Burial Test

In accordance with a previous work by the author^18^ for the biodegradation of extruded PHB and chemically modified PHB in the presence of soil microorganisms, a controlled reactor was employed. The experiments were carried out in an aerobic environment at 25 ± 1 °C, under the controlled moisture of approximately 55%.

Specimens of two different types were applied in the biodegradation test. The first comprised an object for tensile testing purposes (a dumbbell shape) to a given standard,^19^ while the other was the test specimen of size 2 × 2 cm for fluorescence microscopy, see section 2.11). Samples of the films were placed in the soil (a mixture of 650 g perlite and 8 L of soil), such that minimum layers above and below the samples were 4 cm deep and the horizontal spacing between the samples was at least 1 cm. Perlite (a heat expanded aluminosilicate) was added to aid aeration of the soil and retain water. A flow of moistened air was supplied at the base of each vessel every 2 h for 15 min. Observations were made at the beginning and end of the test relating to the dry matter of the soil and the value for soil exchange capacity (pH_KCl_), so as to check on the process. At the close of the soil burial experiment it was discerned that the moisture level equaled ca. 55% and pH_KCl_ ca. 7.2.

The samples of the extruded PHB and chemically modified PHB films were removed from the soil after 40, 60, and 90 days, brushed softly, washed with distilled water several times, and dried at laboratory temperature until a constant weight was obtained. Afterward, the samples were weighed to the precision of five decimal places, and change in this parameter for the samples was denoted as normalized weight loss according to eq 4, where *w*_O_ was the weight of the sample prior to commencing the test (g) and *w*_D_ constituted the weight after 40, 60, or 90 days of testing (g).
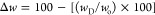
4

A visual assessment followed, along with morphological analysis by scanning electron microscopy, polarized optical microscopy, XRD, and DSC.

### 2.5 Characterization of the Films by Polarized Optical Microscopy (POM)

The crystal morphology and interference colors of the extruded PHB and chemically modified PHB films were characterized by polarized light microscopy (PLM; Nikon Eclipse 50i; Nikon, Japan). The scale bar was created within ImageJ software, version 1.5 (W. Rasband, National Institutes of Health, United States).

### 2.6 Scanning Electron Microscopy

The morphology of the extruded PHB and chemically modified PHB films was studied on a scanning electron microscope (a Phenom Pro X device equipped with the Pro Suite; Phenom-World BV, Eindhoven, Netherlands), set to the magnification of 1000× and acceleration voltage of 15 kV.

### 2.7 Differential Scanning Calorimetry

A total of 5 ± 1 mg of a dried sample was sealed in standard aluminum cells and than were analyzed on a Mettler Toledo DSC1 STAR calorimeter across a temperature range of −30 to 200 °C at the heating rate of 10 °C.min^–1^. The DSC system was purged with nitrogen, and transition temperatures (e.g., *T*_g_ or *T*_m_) for all the samples were determined from the second heating scan. The degree of crystallinity (*X*_c_, %) of the PHB materials was calculated according to eq 5, where Δ*H*_m_ is the enthalpy of melting of the samples, Δ*H*_0_ constitutes the same for 100% crystalline PHB (146 J.g^–1^) and *w* is the weight of the sample.^20^
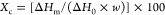
5

### 2.8 Attenuated Total Reflectance Infrared Spectroscopy

FTIR spectra for the extruded PHB and chemically modified PHB films were recorded on a FTIR Nicolet iS10 unit (Thermo Fisher Scientific, U.S.A.) fitted with an ATR Smart MIRacle adapter containing a diamond crystal. Such analysis comprised 64 scans at wave numbers ranging from 4000 to 500 cm^–1^ and a spectral resolution of 4 cm^–1^. The data gathered were evaluated in Omnic 8 software (Thermo Fisher Scientific, U.S.A.).

The polymer samples were analyzed after drying, and FTIR spectra were taken at five different points on the surfaces of the films. For the quantitative analysis conducted, each of the five spectra was normalized and curve-fitted in Omnic 8 software (Thermo Fisher Scientific, U.S.A.); the area (*A*) for each band found by curve fitting was integrated by the software. The value for carbonyl index (*I*_C=O_) was calculated from the ratio of such areas (*A*) under carbonyl (C=O) bands at 1720–1740 cm^–1^ for PHB (eq 6), while crystallinity (*I*_C–O_) was determined from the ratio of areas at 1230 and 1453 cm^–1^ (eq 7).^14,21^
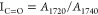
6
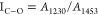
7

### 2.9 X-ray Diffraction Analysis (XRD)

Wide-angle, X-ray diffraction analysis involved the use of a Rigaku Miniflex 600 unit (Rigaku Corporation, Japan). Co K_α_ radiation was Ni-filtered, and scans (5° 2Θ·min^–1^) were performed in the reflection mode in the 2Θ range from 5 to 50°.

### 2.10 Gel Permeation Chromatography (GPC) Measurements

Samples (5 mg) were dissolved in chloroform (1 mL) at 70 °C and filtered through 0.45 μm polytetrafluoroethylene (PTFE) syringe filters. GPC was performed in a 185 Agilent HPLC series 1100 chromatograph (Santa Clara, CA 95051, United States) with a PLgel mixed-c 5 μm, 7.5 × 300 mm column, with chloroform as the mobile phase. Twelve polystyrene standards (0.2–2000 kDa) were used for calibration.

### 2.11 Fluorescence Microscopy (Live/Dead Bacteria)

Immediately after sampling of the films from a soil environment, the samples were rinsed by distilled water, placed on a slide, and stained with a solution of laboratory-prepared fluorescent dye (a combination of SYTO9 dye and propidium iodide) for 30 s. After any excess dye had been removed, the specimens were rinsed once with distilled water and then studied on an Olympus BX53 fluorescence microscope (Olympus, Japan). SYTO9 has an excitation maximum of 480 nm and propidium iodide is evident at 490 nm. These wavelengths were observed with the aid of a SYTO9 dye excitation filter no. 3 and propidium iodide dye excitation filter no. 5. Microorganisms labeled with SYTO9 dye were denoted as alive (green), whereas propidium iodide was flagged as dead (red). The resultant images were analyzed in cellSens software.

### 2.12 Sequence Analysis of Microbial Communities Present during Biodegradation

Portions of the extruded PHB film, chemically modified PHB films and blank soils were obtained during the course of the soil burial test. DNA was isolated from the surfaces of the materials, and a DNeasy PowerSoil DNA extraction kit (Qiagen USA) then used to amplify specific regions of the rRNA genes of fungi ITS2 (18S) and bacteria V3–V5 (16S) by utilizing the primers F357 (5′-CCTACGGGAGGCAGCAG-3′) and R926 (5′-CCGYCAATTYMTTTRAGTTT-3′), or ITS3F (5′-GCATCGATGAAGAACGCAGC-3′) and ITS4R (5′- TCCTCCGCTTATTGATATGC-3′), respectively, with barcoding and the universal overhang. Illumina sequencing adaptors were introduced in the second PCR, all in accordance with the given instructions.^22^ The products were evaluated by agarose electrophoresis, quantified with a fluorimetric, high sensitivity Acugreen kit (Bioline) and pooled into a library. Sequencing took place on a MiSeq unit (Illumina) running the reagent kit v2 and paired-end 250 nt reads in an external laboratory (SEQme s.r.o., Czech Republic). The data were further processed with the DADA2 R package^23^ and visualized by the phyloseq R package^24^ and ComplexHeatmap^25^ R packages. Taxonomy was assigned for the bacteria according to the SILVA 132 SSU NR 99 reference database^26^ and the 8.3 release of the UNITE reference database for fungi.

## 3 Results and Discussion

All the designed formulations (Table 1) were able to undergo processing by melt blending and thermocompression. The resultant films differed in macroscopic and microscopic appearance, depending on the concentration of the free-radical initiator (L) and the ratio of it to MA.

### 3.1 Characterization of the Chemically Modified PHB

The FTIR spectra for the extruded PHB and chemically modified PHB films show characteristic absorption bands for PHB at 2800–3200, 1700–1750, and 600–1500 cm^–1^ (Figures S1 and S2). Ma et al.^27^ state that PHB (aliphatic polyester) exhibits a strong FTIR absorption band at around 1720 cm^–1^, which represents the stretching vibration of carbonyl groups in the crystalline region of PHB, as well as a shoulder at 1740 cm^–1^ assigned to another for such groups in its amorphous region.^27^ The absorption band at ca. 1380 cm^–1^ relates to the vibration (symmetric wagging) of CH_3_ groups, while those at 1180 and 1130 cm^–1^ are characteristic of asymmetric and the symmetric stretching vibrations of the C–O–C group, respectively.^28^ Absorption bands at 980, 1230, and 1275 cm^–1^ indicate the crystalline phase of PHB with that at 1181 cm^–1^ assigned to the amorphous phase. It is proposed that the occurrence at 1230 cm^–1^ pertains to the conformational band of the PHB helical chains.

As for maleic anhydride (MA), two characteristic absorption bands are evident at 1780 and 1850 cm^–1^, corresponding to the symmetric and asymmetric stretching of the carbonyl groups of cyclic MA, respectively (spectra not presented).

The FTIR spectrum of the chemically modified PHB closely resembled that of the extruded PHB (Figures S1 and S2), probably since they were nearly identical chemically (see Table 1).^14^

It has been reported^29^ that the thermal degradation of PHB by six-membered ring transition states gives rise to the formation of carboxylic acid and unsaturated groups; evident herein via the emerging bands at ca. 3437 and 1629 cm^–1^ that denote an increase in the number of hydrogen-bonded groups and C=C bonds, respectively. Thermal degradation also causes a decrease in crystallinity, which in turn leads to a reduced intensity in crystalline bands at ca. 1724 and 1230 cm^–1^. The FTIR spectra for the films do not present an absorption band at 1629 cm^–1^, only a pseudo micro one at 3436 cm^–1^. This suggests that just minimal thermal degradation occurred during preparation of the films, with negligible influence on the course and degree of biodegradation.

Quantitative FTIR analysis revealed that no significant alteration in the crystallinity of PHB was brought about by chemical modification or thermal degradation.^29^ The carbonyl index (*I*_C=O_) for extruded PHB equaled 8.32 ± 1.8, whereas values for the chemically modified PHB ranged between 7 and 10 with great variability in standard deviation. The crystallinity indices (*I*_C–O_) for them were 2.19 ± 0.28 and 2.06–2.57, respectively. These findings were in agreement with those of the DSC analysis conducted (Table 3), demonstrating that no statistically significant difference existed in the degree of crystallinity (*X*_c_). Hence, the conclusion was made that the techniques employed to modify PHB chemically did not exert a significant effect on the crystallinity of the prepared films.

**Figure 1:**
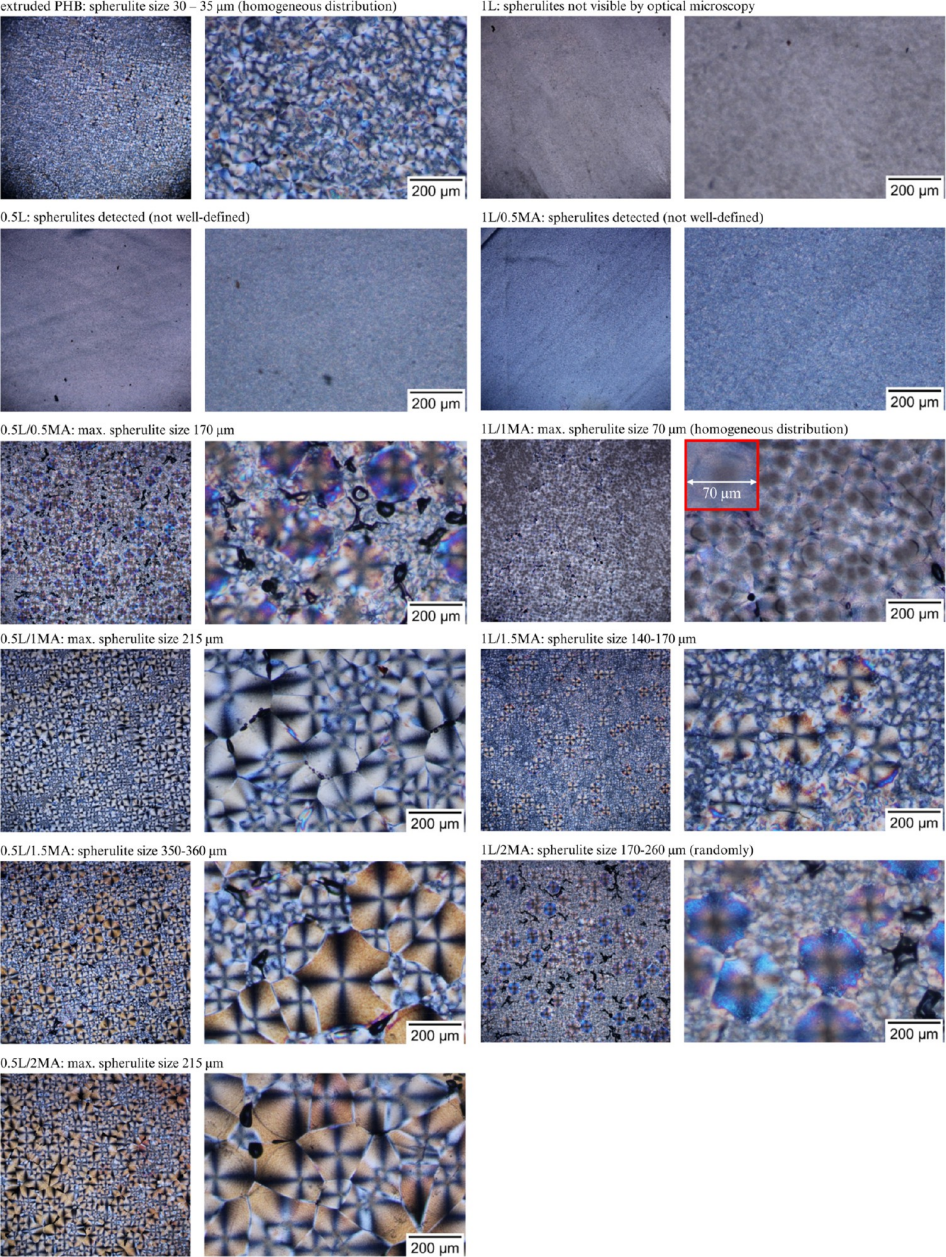
Polarized optical micrographs of the extruded PHB and chemically modified PHB films (left, 4× magnification; right, 10× magnification).

Figure 1 contains a series of optical micrographs of the extruded PHB and chemically modified films. The various interference colors obtained by POM in Figure 1, highlight the differences between the crystalline morphologies of the compared samples. Since all the materials had been synthesized under similar conditions regarding temperature and processing, it was the composition of the prepared polymeric layers that informed the resulting structures, the latter possessing typical forms of cold crystallization, banded and nonbanded spherulites.^30,31^ The thickness of every film was comparable, so this factor had not influenced matters and could be disregarded, in agreement with the literature.^32^ Change in the interference colors observed was gauged by retardation, calculated by birefringence (Δ*n*) multiplied by the thickness of the sample (*d*), in accordance with other authors.^33,34^ The relationship between interference color and retardation is described by resources, such as the Michel-Levy chart, and applying it showed a rise in birefringence interfaces in the volumes of the compared systems (Figure 1). The value for the boundary retardation of the observed colorful spherulite interfaces was 300, and exceeding it led to an increase in the number of such spherulites observed by POM.^30,33,35^

Optical microscopy (Figure 1) permitted analysis of microspherulitic and macrospherulitic structures in the samples, and the predominance of either related to the overall intermolecular interactions that occurred between the PHB, free-radical initiator, and MA, in turn significantly modifying crystal growth processes. The presence of ring-banded spherulites is generally attributed to the periodical lamellae twisting, along the radial growth direction of the spherulites. A circular spherulitic structure with a characteristic Maltese cross was discerned herein, and the diameters of the spherulites clearly related to the content of MA. Dense, fine crystals were seen in the crystal morphology of the PHB sample treated with the free-radical initiator but without MA (Figure 1). In the case of 1% of the initiator, spherulitic-like objects were observed, referred to in the literature as pseudo spherulitic structures.^36^ The assumption was made that the free-radical initiator acted as a cross-linking agent, similarly to Najafi et al.,^37^ a phenomenon whereby the network obstructs the packing of polymer chains, causing cessation of spherulite growth.

The results of POM supported the primary aim of this work, i.e., devising a polymer modification reaction that would give rise to various perfect or imperfect crystals, their forms depending on the given chemical composition.

### 3.2 Biodegradation Experiment

The primary mechanism of degradation of some polyhydroxyalkanoates (PHAs) is hydrolysis, catalyzed by temperature, followed by bacterial attack on the fragmented residues.^38^ A number of studies have been performed assessing biotic and abiotic hydrolytic degradation of PHB in different conditions. It should be noteworthy that most publications report hydrolytic studies performed at the physiological (37 °C) and elevated (58 °C, 70 °C) temperatures under variable pH. It was found that hydrolytic degradation of PHB effectively occurs when at strong alkaline pH. For example, Tarazona et al.^39^ investigated the abiotic hydrolytic degradation of PHB in different environmental- and physiological-like conditions of variable pH. They confirmed that for the hydrolytic degradation of PHB materials, very harsh conditions (pH = 12.3) are necessary.^39^ In their work, Bonartsev et al.^40^ stated that the PHB with the relatively high molecular weights are stable against hydrolytic degradation (at 37 and 70 °C, phosphate buffer) for the period of 91 days.^40^

In conclusion, current research^39−41^ suggests that the abiotic hydrolysis of PHB under environmentally relevant conditions (typically not exceeding 30 °C in temperature) to be an extremely slow process, takes several months. Given these insights, the authors assume that the microbial degradation process of PHB represents the primary mechanism of PHB degradation within soil environments, as it occurs significantly more rapidly than simple hydrolytic degradation. Consequently, this abiotic process can be overlooked when evaluating the kinetics of PHB biodegradation under environmentally representative conditions.^39−41^

The course and degree of biodegradation of the films was investigated in two ways. The first was a long-term respirometric test (9 months) performed in closed biometric bottles, but it proved unsuitable for obtaining the required amount of the specimens. For this reason, the decision was taken to carry out a soil burial test over a three-month period. The material broke up when it was handled upon completion of the experiment, and this fragility meant that further determination by XRD and DSC was not possible. In fact, the test specimens had been so affected by the action of soil microorganisms that the planned mechanical tests had to be canceled, too.

#### 3.2.1 Long-Time Respirometric Test

**Figure 2:**
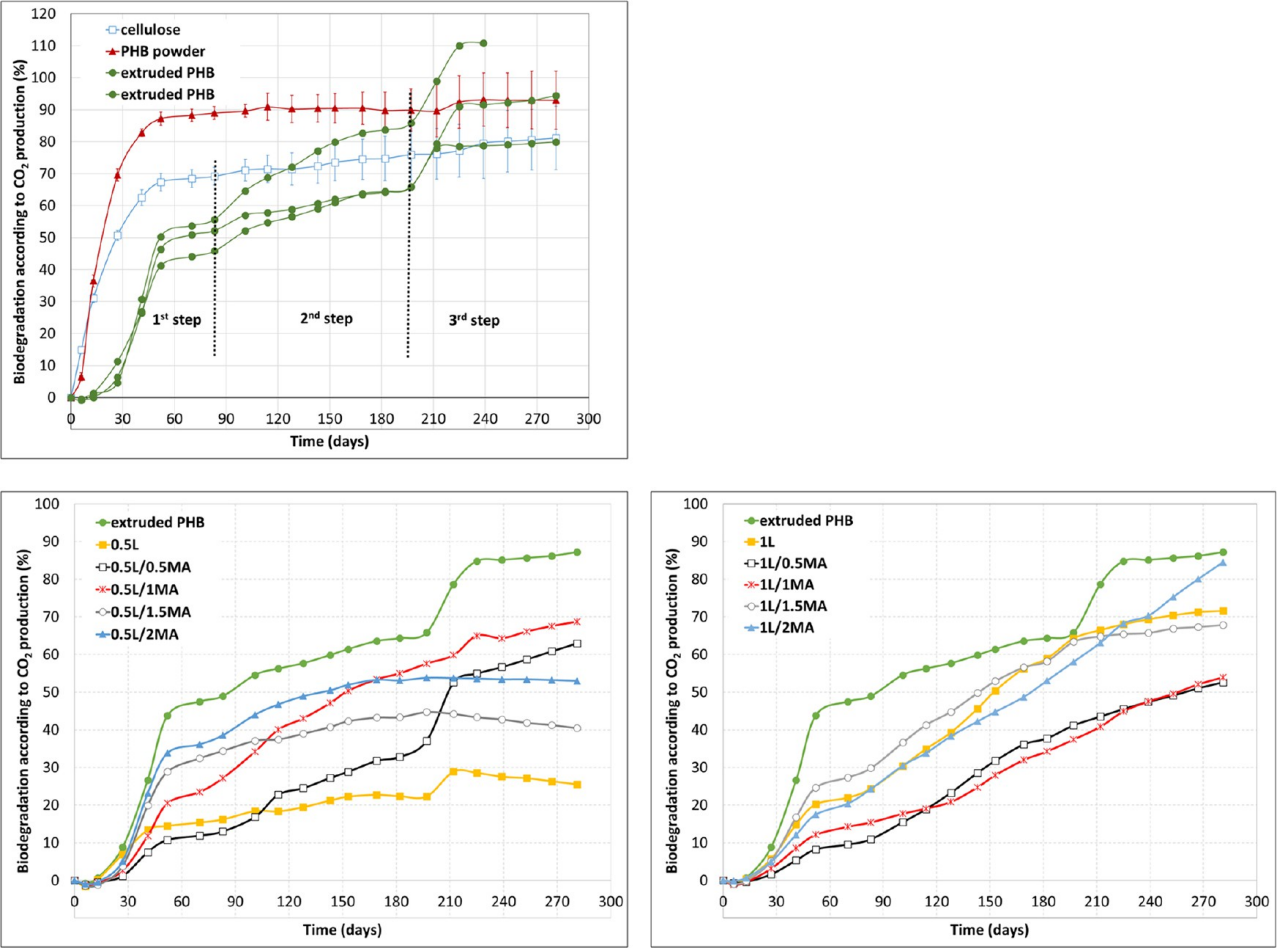
Biodegradation curves of the long-term respirometric test at 55% soil humidity and 25 °C for PHB in powder, extruded, and chemically modified forms.

As seen in Figure 2, the biodegradation curve (dependence on time of carbon mineralization in relation to the CO_2_ generated) for PHB powder was normal: a series of steps typical for the pure polymer in this form. Indeed, it began to degrade almost immediately upon commencement of the experiment. No lag phase was observed for it, indicating very good utilization of the polymer by microorganisms. The powder exhibited an extremely high degradation rate from the very start, achieving 88.96 ± 2.02% mineralization within 90 days.

The following is generally acknowledged: (i) the microbial degradation of crystalline heat-treated polymers proceeds in a selective manner, with amorphous regions being degraded prior to crystalline regions; (ii) the extent of cross-linking (network density) alters the course of biodegradation,^42^ where as a rule the non-cross-linked phase may undergo it more easily; and (iii) the biodegradation (as per loss in weight) of melt-grafted PHB is gradually heightened in parallel with an increase in the graft degree of maleated PHB, while the graft phase is preferred by microorganisms.^13^ These factors can initiate a multistage process of microbial degradation, and three such stages transpired herein for the extruded PHB and chemically modified PHB films.^43,44^ This finding was in agreement with García-Depraect et al.,^44^ although they aimed to expand on the standard biodegradation test method applied by adding detailed analyses of kinetics, carbon fate, and the effect of particle size and did not investigate why the biodegradation curve of PHB took place over three stages.

Figure 2 shows that the first stage of biodegradation for all the studied films commenced during the first 90 days of the test, while the third and last stage started after ca. 200 days, as evidenced by the samples of extruded PHB, 0.5L and 0.5L/0.5MA. A slight indication of the latter was also evident for the 1L and 1L/1.5MA films, approximately on day 180.

On the basis of DSC analysis and microscopic observation, it can be stated that the first stage of the biodegradation curve corresponded to the breakdown of the amorphous phase. Figure 2 presents a rising trend related to the amount of MA present and increase in spherulite size (Figure 1). Data suggested that the degree of mineralization in the first stage of biodegradation was not only influenced by MA content (as reported by Chen et al.^13^), but also the availability (for microorganisms) of an amorphous phase in the interlamellar space of spherulites. At the completion of this stage, the PHB films exhibited degradations of 11.87%, 23.48%, 34.42%, and 36.13% for 0.5L–0.5MA, 1MA, 1.5MA, and 2MA, and 9.51%, 15.40%, 20.38%, and 27.33% for 1L–0.5MA, 1MA, 2MA, and 1.5MA, respectively.

The second stage of biodegradation produced indistinct respirometric data (visualized as curves) for the 0.5% L and 1% L series. An assumption was made that crystal structure and spherulite morphology played a role, on the basis of findings in the literature. Tomasi et al.^7^ wrote that the biodegradation rate of PHB decreased as a consequence of a rise in the exactness of the crystalline phase (greater regularity and homogeneity in spherulite morphology). This was consistent with observations for the 0.5L/1.5MA film, which demonstrated the least extent of degradation (in percent) in the second stage, wherein the largest spherulites were detected. However, the data obtained in this study (Figure 2) suggested that this was a much more complex process in connection with heterogeneous microbial cultures. It should be noted that sample 0.5L commenced its second stage of biodegradation after 190 days. Taking into consideration the SEM, POM and DSC analyses, it was concluded that the cause was the reorganization of the crystalline phase, leading to the creation of new spherulitic structures via nucleation (section 3.4), the latter likely influencing the physiological behavior of the soil microorganisms and retardation of the biodegradation process.

**Figure 3:**
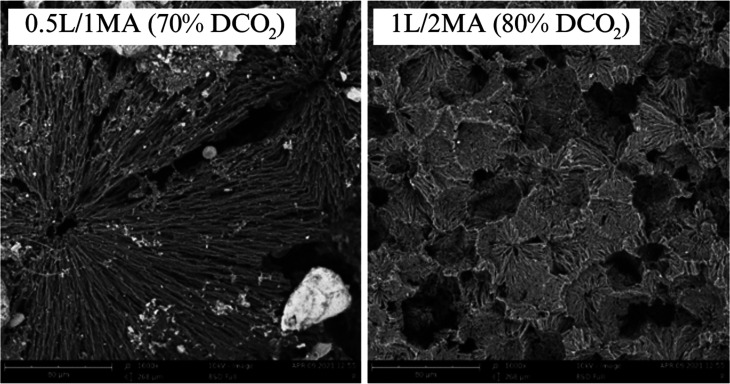
SEM images (scale: 80 μm) of the chemically modified PHB films after 240 days of biodegradation in the long term respirometric test (at 55% soil humidity and 25 °C).

The processes taking place within the third stage of decomposition were difficult to interpret from the respiratory curves. The supposition was that total destruction of the crystalline structure occurred with associated microfragmentation of the crystalline phase, which subsequently underwent biological decomposition. In order to confirm this hypothesis, the soil environment was investigated by optical microscopy. After 240 days of the respiratory experiment, the samples and microfragments that biodegraded over the course of three stages were found to be completely absent from the given environment, however, materials demonstrating two such stages were still evident. Microfragments of these discerned by microscopy were subsequently isolated and analyzed by SEM, as detailed in Figure 3. The images revealed the presence of a crystalline structure, even though a high percentage of decomposition in connection with the amount of CO_2_ produced was determined for the samples (e.g., 1L/2MA: ca. 80% mineralization; 0.5L/1MA: ca. 70%).

#### 3.2.2 Soil Burial Test: Macroscopic Observation and Gravimetry

Figure S3 shows the appearance of the surfaces of samples prior to the burial test and during it at intervals of 40, 60, and 90 days. All the films had turned milky in appearance by its close through the excretion of metabolites (e.g., acetic or butyric acid) from 3-hydroxybutyric acid.^45^ Significant surface roughness was observed as a result of microbial attack by loosely and strongly associated microorganisms. The timeline gauged by SEM revealed that biological erosion took place preferentially on the surfaces of the PHB films, which demonstrated a tendency toward porosity and deterioration in shape. Such erosion probably arose through bacterial attack (as anticipated) and/or typical fungal (enzymatic) degradation in a soil environment. Woolnough et al.^46^ reported that rapid loss in weight by PHB in garden soil was associated with numerous, diverse soil microorganisms capable of degrading it.

The percentage in dry weight loss of each film was plotted against time (duration of the soil burial test), as displayed in Figures S4 and S5. It was found that the extruded PHB film lost 95.7% of its original weight after 3 months. Several publications describe a significantly slower course of PHB biodegradation in soil, though. For example, dumbbell-shaped extruded PHB samples (injection molded and of unknown thickness) reduced by 7% from their original weight after approximately 7 months in clay soil at 28 °C.^47^ Boyandin et al.^48^ stated that PHB (cast film, 0.1 mm thick) showed 16% loss in weight during field biodegradation tests in the summer in Russia. Avella et al.^49^ wrote about the weight reduction of thermocompressed PHB (1 mm thick samples) in garden earth, finding it had dropped by 11% after 6 months at 23 °C. Mousavioun et al.^50^ investigated extruded PHB films with a thickness of 100 μm in a year-long soil burial test (under environmental conditions), which decreased by 45% in weight in this period. Such variation in findings in the literature can be explained by differences in the thicknesses of the sample sets and processing methods employed, giving rise to divergence in polymer surface area, bulk density, crystallinity, crystal structure, and spherulite morphology. Consideration should also be given to the fact that the experiments by the research teams were carried out under different environmental conditions (type of soil, climate). The various molecular weights of the PHB in the aforementioned studies could also have contributed to this divergence in results.

Taking into account the error in measurement experienced in gravimetry caused by a high degree of defragmentation, the conclusion was made that the results of the soil burial test aligned with those of the respirometric experiment.

Previously, it was demonstrated that biological process significantly affects the molecular weights of PHB.^51,52^

In order to verify this fact, the molecular weights (*M*_n_, *M*_w_, *M*_*z*_) of unprocessed PHB (powder) as well as of extruded PHB before and after biodegradation have been evaluated by GPC. The results are listed in Table 2. According to GPC data, *M*_n_ and *M*_w_ of unprocessed PHB are significantly higher than those of the extruded sample, indicating that thermal degradation caused the molecular weight to decrease during processing. This degradation is in good agreement with previously published results for PHB and is likely caused mainly by the random chain scission.^53,54^

**Table 2:** Molecular Weight Change of the Unprocessed PHB (Powder) and Extruded PHB Film Throughout Biodegradation^a^

sample code	*M*_n_ (×10^3^) g mol^–1^	*M*_w_ (×10^3^) g mol^–1^	*M_z_* (×10^3^) g mol^–1^	*D*
unprocessed PHB	87.91	437.9	1350.0	4.981
after biodegradation test				
extruded PHB	41.76	148.5	322.2	3.557
40 days	37.50	125.8	269.8	3.354
60 days	55.58	142.4	270.4	2.563
90 days	51.25	135.1	252.5	2.636

a *M*_n_, number average molecular weight; *M*_w_, weight average molecular weight; *M*_*z*_, *z*-average molecular weight; *D*, dispersity index.

**Figure 4:**
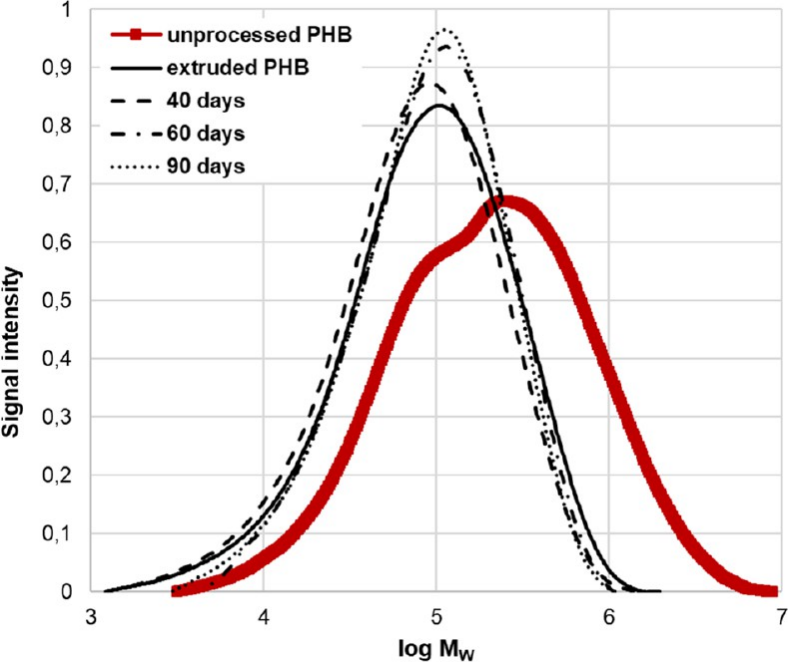
Molecular weight distribution curves of unprocessed PHB (powder) and extruded PHB (film) throughout biodegradation in soil environment (55% soil humidity, 25 °C).

Figure 4 shows the changes in molecular weight distributions with biodegradation time for the extruded PHB. It can be seen from Figure 4 that there is a change in molecular weight distribution with time. The molecular weight distribution curve for extruded PHB after 40 days of biodegradation shifts toward left, indicating the formation of low-molecular-weight compounds.^55^ This change is accompanied by a reduction in the molecular weight of PHB (*M*_w_: from 148.5 × 10^3^ to 125.8 × 10^3^). It is also clear that the action of microorganisms is only on the surface of the polymer. After 60 days of biodegradation, the molecular weight distribution curve for the extruded PHB shifts toward the right, indicating that the low-molecular-weight fragments present in the thermal degradation products are being eliminated or utilized.^55^ This change is accompanied by a slight increase in the molecular weight of PHB (*M*_w_: from 125.8 × 10^3^ to 142.4 × 10^3^). The authors believe that initially (after 40 days), soil microorganisms can only utilize a minor fraction of the thermal products, shifting the molecular weight distribution curve slightly toward higher fractions.^55^ After 60 days, a major high-molecular fraction of PHB started to be utilized, shifting toward the right-hand side of the molecular weight scale. These results indicate that during the first 90 days of biodegradation, soil microorganisms can utilize chain-end low-molecular-weight compounds and are unable to perturb high-molecular-weight fractions.^52,55,56^

In accordance with Park et al.^52^ it was confirmed that soil microorganisms biodegraded PHB and that the low molecular weight fractions of PHB tended to biodegrade more rapidly than high molecular weight fractions.

### 3.3 Morphological Changes: Scanning Electron Microscopy

**Figure 5:**
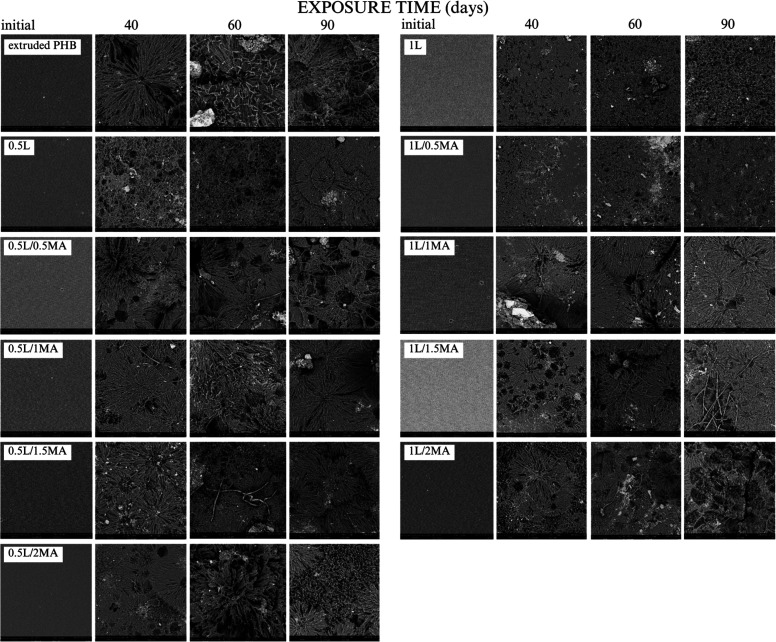
SEM images (scale: 80 μm) of the extruded PHB and chemically modified PHB films before and after specific periods of biodegradation in the soil environment (soil burial test, 55% soil humidity, 25 °C), and brief descriptions of the samples prior to biodegradation, as discerned by optical microscopy.

The crystalline morphology apparent in the extruded PHB and chemically modified PHB samples, following biodegradation of the amorphous phase on their surfaces, showed the initial persistence of lamellar ribbons (Figure 5), confirming rapid, initiatory erosion of the amorphous region, as proposed in the section on the respirometric test. Other authors^2^ presumed that such preferential degradation of amorphous regions arose through surface-level homogeneous enzymatic action by diffused extracellular depolymerases, as well as degradation by considerable, localized enzymatic action with colonization by degrading bacteria. Alteration in the crystal structure of PHB, therefore, probably affected the colonization or physiological action of the degrading bacteria on the surfaces of the materials. In addition to erosion of the amorphous regions, evidence existed of degradation of lamellae. SEM images revealed the formation of spherical holes at the centers of crystals and on boundary lines. A circular pattern of degradation was apparent in the middle of spherulites. Considering the course of biodegradation of the chemically modified PHB over time, changes in crystal structure clearly influenced the size, number, and formation of the spherical holes. This phenomenon was previously reported by Tomasi et al.,^7^ Nishida and Tokiwa,^2^ and Morse et al.,^57^ their explanation being that imperfection in lamellae packing near nucleation points led to preferential enzymatic attack of the centers, giving rise to the holes observed. As degradation continued, these holes spread and penetrated the film, leaving behind only spherulitic remnants (Figure 5), suggesting the central parts of spherulites eroded the most rapidly. Ring-shaped remnants have also been reported for anaerobically degraded samples of the microbial copolymer poly(3-hydroxybutyrate-*co*-3-hydroxyhexanoate)^57^ and films of enzymatically degraded melt-crystallized poly(butylene adipate).^9^

**Figure 6:**
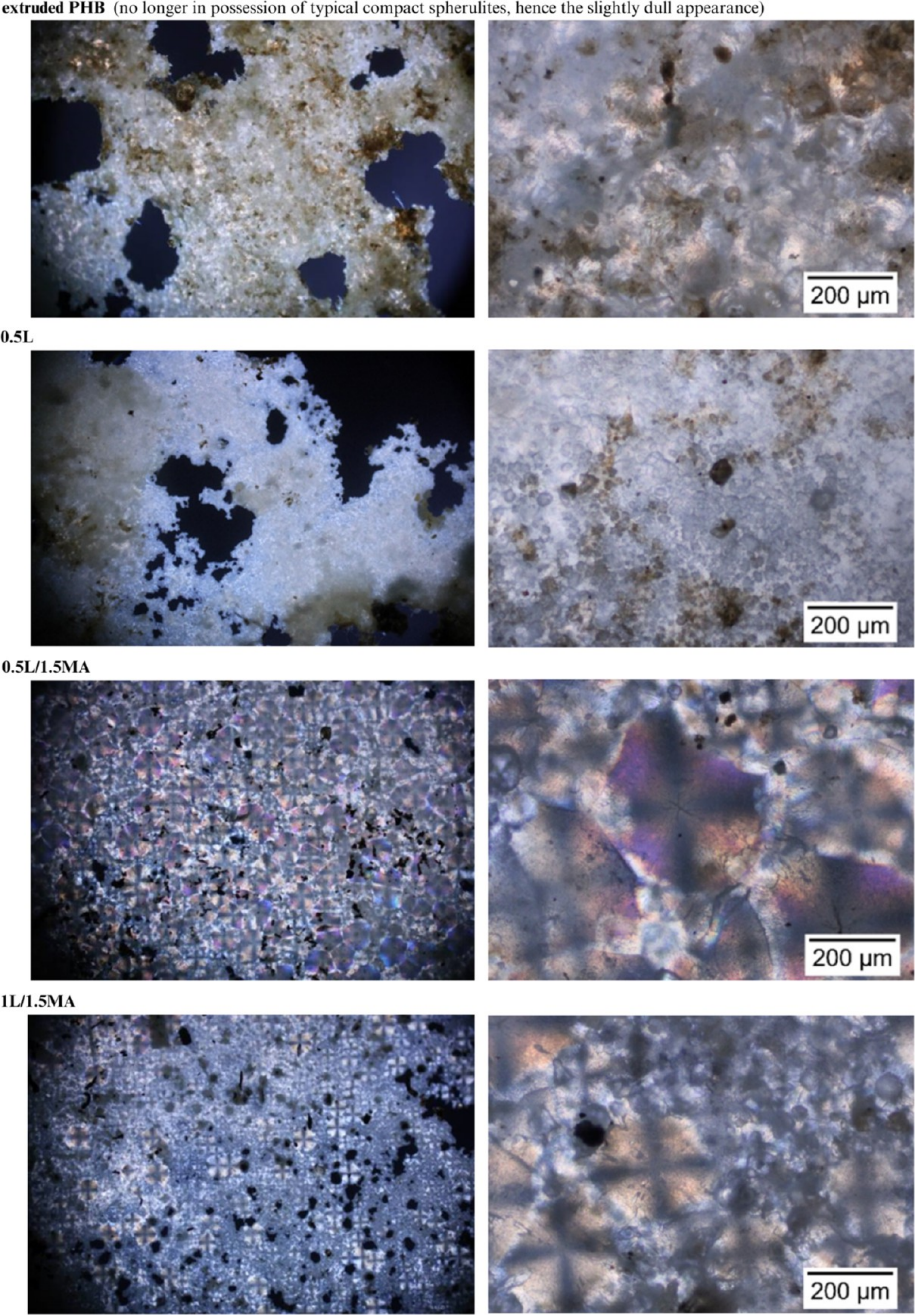
Polarized optical micrographs of the extruded PHB and selected chemically modified PHB films after the 90-day biodegradation soil burial test (left, 4× magnification; right, 10×); the black/white appearance in some instances typifies retardation of less than 300; the thickness of degraded samples decreases significantly, and a concurrent decrease is seen in the volume representation of birefringence interfaces.

Based on thorough examination herein of SEM and POM images (Figures 5 and 6, following biodegradation), the authors concluded that microorganisms potentially preferred small spherulites over larger ones when decomposing the crystalline phase. In this context, the POM images (Figure 6) contained far fewer colorful spherulitic interfaces.

The PHB samples treated with the free-radical initiator but absent of MA, upon biodegradation of the amorphous phase, possessed an exposed, underlying crystalline morphology, wherein the 0.5L film exhibited finer, dense crystals while a pseudo spherulitic structure was observed for the 1L specimen. After 90 days of biodegradation, a new spherulitic structure began to emerge for the 0.5L sample, in accordance with morphological features evident in polarized optical micrographs. It was hypothesized that the nucleation process constituted the cause, and DSC results for the partially degraded 0.5L film subsequently confirmed this assumption (see section 3.4).

### 3.4 Differential Scanning Calorimetry

DSC analysis was carried out to discern potential change in the thermal events of the PHB materials induced by soil microorganisms during biodegradation. Table 3 details the resultant data in terms of their temperatures for melting point (*T*_m_) and crystallization (*T*_c_), as well as crystallinity (*X*_c_) before and after the biodegradation experiment, as obtained from second heating runs (melt crystallization). The data merely pertain to samples exposed to the given environment for 0, 40, 60, and 90 days (the soil burial test), after which time the associated fragments contained numerous soil particles and any results from DSC would not have been relevant.

**Table 3:** Data Obtained from DSC Thermograms in the Second Heating Scan^a^

	*X*_c_ (%)				*T*_1m_ (°C)				*T*_2m_ (°C)				*T*_c_ (°C)			
		exposure time (*d*)				exposure time (*d*)				exposure time (*d*)				exposure time (*d*)		
sample	initial	40	60	90	initial	40	60	90	initial	40	60	90	initial	40	60	90
extruded PHB	55.35 ± 2.61	50.91	66.50	72.40	169.6 ± 0.54	169.89	169.49	168.24	174.6 ± 0.21	174.52	173.26	173.93	74.55 ± 1.02	90.77	91.65	87.09
0.5L	51.18 ± 1.49	52.39	64.72	55.54	166.9 ± 1.43	164.77	164.06	161.75	168.7 ± 0.72	171.73	171.02	170.35	103.8 ± 2.03	93.24	90.73	86.18
0.5L/0.5MA	50.38 ± 2.71	62.46	59.22	61.39	162.9 ± 0.41	161.56	161.69	161.8	171.5 ± 0.17	171.11	170.73	171.17	83.83 ± 0.73	85.82	86.34	84.28
0.5L/1MA	53.55 ± 0.91	52.51	64.34	62.34	164.5 ± 0.95	160.52	160.15	160.14	172.0 ± 0.32	170.49	169.75	169.47	88.80 ± 0.79	86.51	85.85	83.11
0.5L/1.5MA	53.76 ± 0.79	52.55	53.36	62.07	161.1 ± 0.75	160.17	159.68	157.96	170.5 ± 0.94	170.46	170.13	168.71	63.48 ± 4.69	84.39	83.21	79.99
0.5L/2MA	53.00 ± 0.78	65.43	63.19	64.95	160.1 ± 0.93	157.75	155.91	155.32	169.6 ± 0.14	168.73	167.50	166.81	59.67 ± 1.86	79.89	74.47	69.77
1L	50.62 ± 4.30	49.13	61.31	62.23	160.5 ± 1.34	160.82	157.56	159.58	169.7 ± 0.33	167.73	166.53	167.41	93.99 ± 16.2	103.61	97.98	99.70
1L/0.5MA	47.77 ± 1.11	49.50	55.47	61.69	158.8 ± 0.36	158.51	156.97	157.23	168.9 ± 0.19	168.81	167.30	167.72	87.94 ± 1.17	87.42	86.76	84.23
1L/1MA	51.41 ± 0.92	50.25	59.28	62.77	157.7 ± 0.44	157.74	156.9	156.64	168.7 ± 0.12	169.11	168.62	167.71	66.76 ± 0.57	75.01	79.84	75.67
1L/1.5MA	49.16 ± 1.70	49.98	63.96	60.86	157.1 ± 0.23	157.92	155.92	155.44	168.5 ± 0.34	169.02	167.58	167.15	65.52 ± 2.80	77.48	72.3	74.31
1L/2MA	52.62 ± 2.43	48.64	61.37	64.99	158.8 ± 0.94	157.27	155.52	155.63	168.5 ± 0.36	168.49	167.24	166.79	58.45 ± 0.14	76.3	77.19	75.81

a Degree of crystallinity, *X*_c_; Melting point temperature, *T*_1m_ for the chemically modified phase and *T*_2m_ for PHB; Crystallization temperature, *T*_c_, average ± standard deviation, *n* = 3. Standard deviation for the biodegradable samples is ca. 1.8% for *X*_c_, 0.76 °C for *T*_1m_, 0.35 °C for *T*_2m_, and 2.91 °C for *T*_c_.

#### Before Biodegradation

**Figure 7:**
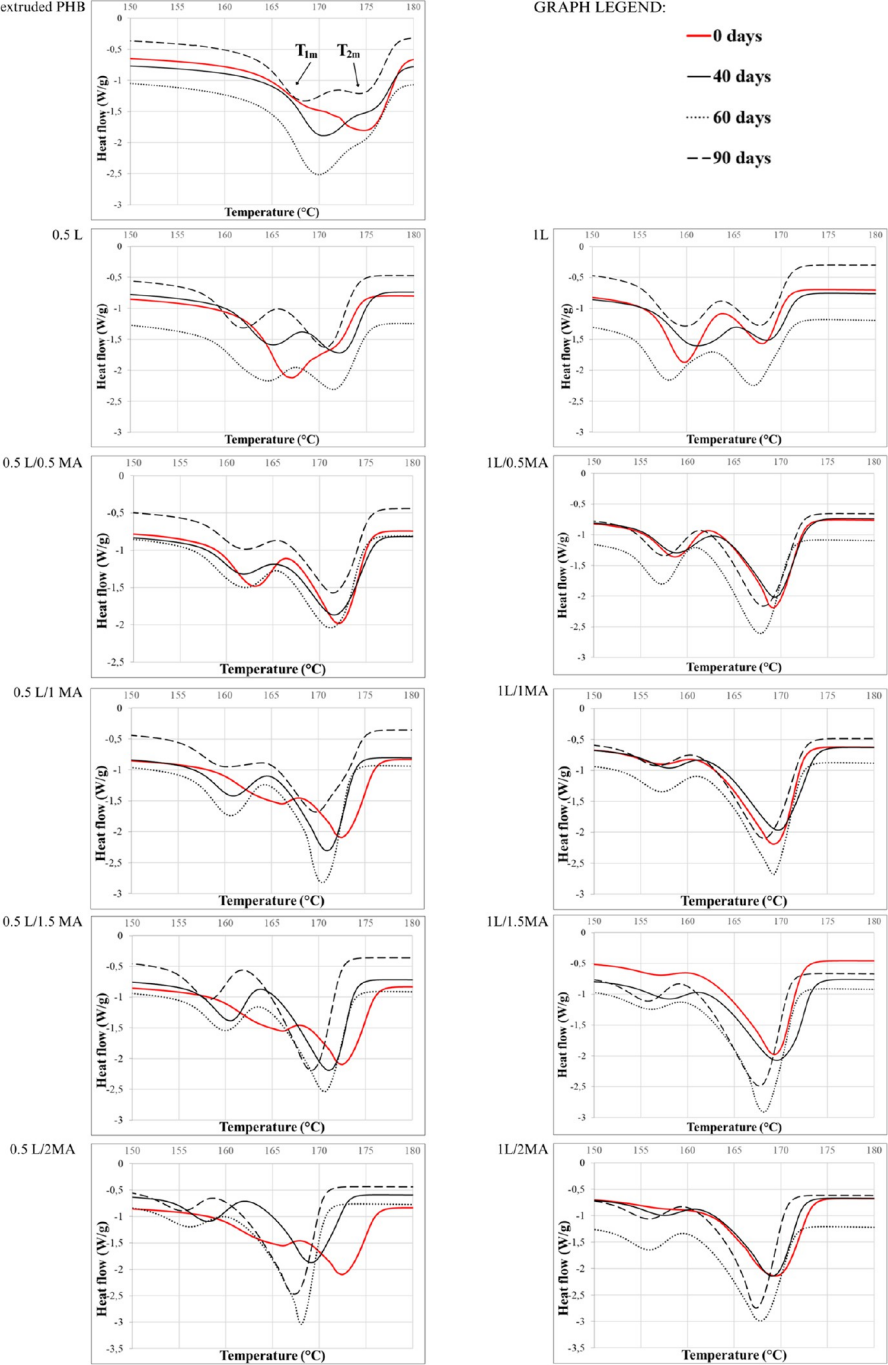
DSC curves recorded during the second heating run for the extruded PHB and chemically modified PHB films before and after specific periods of biodegradation.

A sharp melting peak is observed for extruded PHB at 174.6 ± 0.21 °C (*T*_2m_), with a small shoulder at 169.6 ± 0.54 °C (*T*_1m_) and no discernible glass transition. In agreement with Chen et al.,^13^ DSC melting curves for the chemically modified PHB samples, which experienced various chemical modifications in the second run, possessed multiple melting peaks; the relative areas of two such peaks depended how much they had been modified, i.e., the greater it was in extent, the more pronounced the lower temperature peak (*T*_1m_, chemically modified phase; Figure 7).

Table 3 shows that chemical modification caused a decrease in melting temperature (*T*_1m_, *T*_2m_) and affected *T*_c_, with the exception of the extruded PHB samples. Thus, it was concluded that modifying PHB chemically with the free-radical initiator alone (without MA; 0.5L, 1L) exerted a significant effect on the morphology of PHB, reducing the sizes of crystals or forming less precise ones (pseudo spherulite in structure) that subsequently showed a lower melting point. In the case of PHB treated with the free-radical initiator and MA (L/MA), the change in *T*_m_ and *T*_c_ was in accordance with findings by Chen et al.,^13^ who stated that introducing MA could hinder the crystallization of PHB due to its limited affinity for said process. As a consequence, PHB had the potential to obtain different, imperfect crystals (see Figure 7) during the cooling process. When reheated, such imperfect crystals may melt at a lower temperature, bringing about a shift in melting peak for existing crystals to a lower temperature.^13^

The level of crystallinity slightly diminished by carrying out the above chemical modifications, otherwise only negligible differences were noted between the samples.

#### After Biodegradation

Extruded PHB was relatively regular in spherulitic structure and exhibited the highest rate of biodegradation. Neither *T*_1m_ nor *T*_2m_ altered significantly during 90 days of the test, however the percentage of crystallinity increased by ca. 17%, and the relative areas of the two melting peaks changed in relation to *T*_1m_. These results were in accordance with the SEM and POM observations (Figures 5 and 6), so it was assumed that the original spherulitic structure of PHB had been disturbed within the 90 days. All the PHB samples in the chemically modified series of 0.5% and 1% L showed a slight drop in *T*_1m_ and *T*_2m_ over time (1–2 °C) and a rise in crystallinity (10%). This suggested that during the 90 days of biodegradation, following decomposition of the amorphous phase, a greater extent of disorder affected the crystalline phase, yet the spherulitic structure remained intact (the relative areas of the two melting peaks did not change). This could have happened through thickening of the PHB crystallites, likely caused by a decrease in chain mobility in the amorphous region since the processes were taking place in the soil environment.^58^

It would seem, however, that alteration in the morphology of the chemically modified PHB during the biodegradation experiment was more complex, owing to the various changes which took place. In the case of the 0.5L PHB film, *T*_2m_ (PHB crystals) increased by 3 °C after day 40 and the relative areas of the two melting peaks changed in connection with *T*_2m_ (Figure 7). Within that period *T*_c_ and crystallinity decreased (by 17 °C and 10%, respectively), reflecting the nucleation of PHB crystals during the environmental processes;^59^ in agreement with the SEM (Figure 5) and POM (Figure 6) observations. The explanation for this lies in the plasticizing effect of water that enters the matrix through the eroded surface, disorganizing the crystalline structure of thinner crystallites.^20,60^

### 3.5 X-ray Diffraction Analysis (XRD)

**Figure 8:**
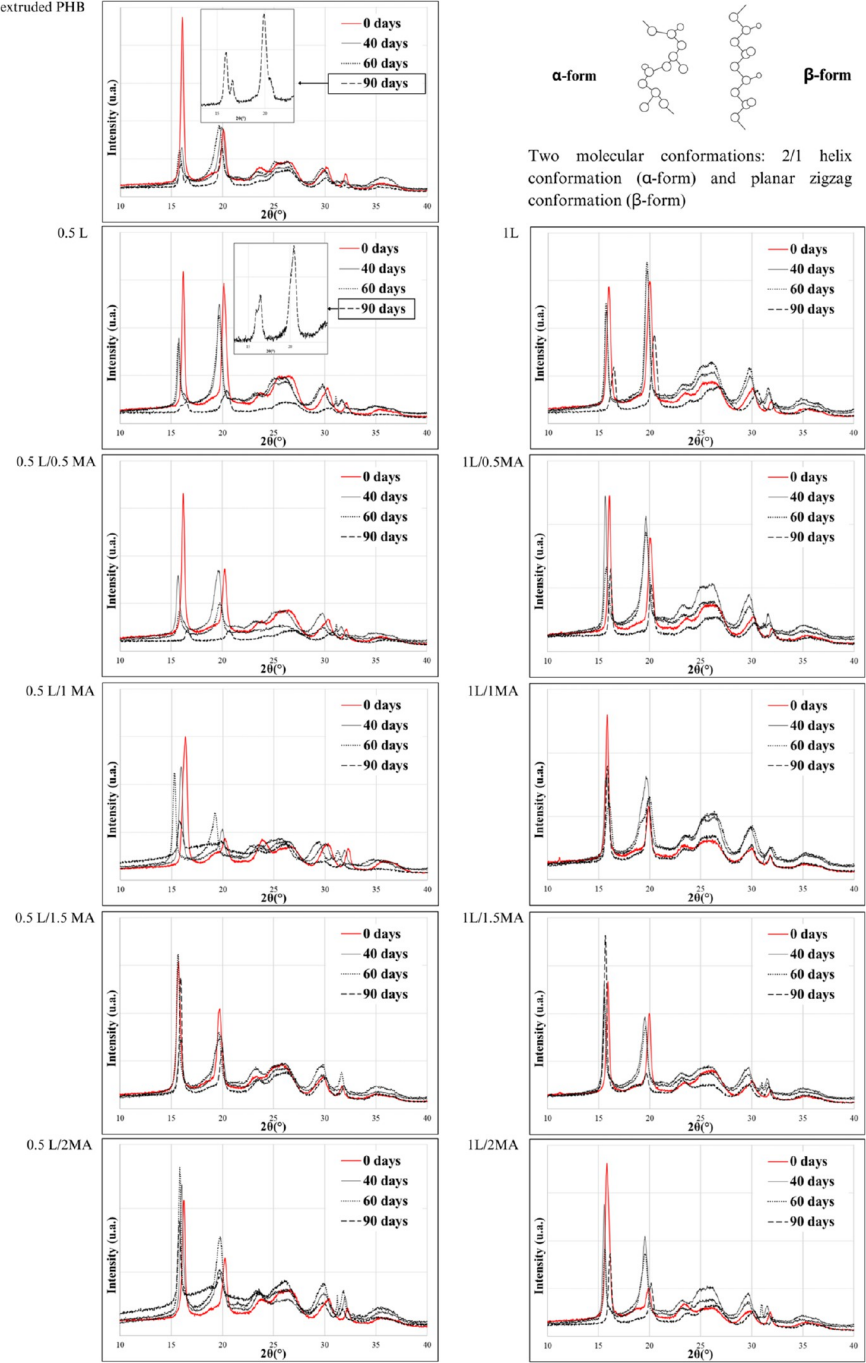
XRD diffractograms of the extruded PHB and chemically modified PHB films before and after specific periods of biodegradation in the soil environment (55% soil humidity, 25 °C).

Figure 8 presents X-ray diffraction patterns for the extruded PHB and chemically modified PHB films before and after 90 days of biodegradation.

#### Before Biodegradation

As described in a work by Quispe et al.,^20^ pure extruded PHB presented a typical α-type structure (left-handed 2/1 helix conformation) due to melting and crystallization processes. They reported that PHB crystallized in an orthorhombic lattice structure. Accordingly, the XRD pattern for PHB presented two strong intensity peaks at 2θ = 16.04° and 20.06° assigned to (020) and (110) planes (α-orthorhombic crystalline structure), respectively. Less intense peaks at 2θ = 25.52° and 26.5° corresponding to (101) and (111) reflection planes were also observed. Signals detected at 2θ = 30.32° and 32.24° were attributed to (130) and (040) planes, respectively. In addition to the presence of orthorhombic α-form crystals with a helical chain conformation, PHB also possesses a small amount of β-form crystals with planar zigzag conformation, as revealed by the diffraction shoulder located at 2θ = 23.86° relating to the (021) plane. This crystalline β-form is characteristic for extruded PHB molded by thermocompression. The presence of β-form crystals indicates a high level of molecular stretching in the amorphous region between the α-crystalline lamellae.^20^

XRD diffractograms (Figure 8) of the chemically modified PHB films resembled those of the pure extruded PHB film, even showing the same reflection peaks, demonstrating that the PHB unit cell had not changed radically. The diffractograms presented peaks that differed as follows: (i) in the intensity of the strong reflection at 2θ = 20.06° due to (110) planes; (ii) in the relative intensities of three reflections between 2θ = 20° and 2θ = 27° that underwent inversion; (iii) in the intensity of reflections at 2θ = 16.04 caused by (020) planes, and at 2θ = 32.24° due to an increase in (040) planes. Such differences are explained thus: (i) by the various degrees of crystallinity,^13,20^ in agreement with the results of DSC analysis; and (ii) by differences in the sizes of spherulites and their preferred orientation effects,^61^ in line with the POM observations. Variation in intensity ratio between the peaks (021) (β-form crystals), (101) and (111) (α-form crystals) was recorded for 0.5L/1MA and 1L/2MA, from which it could be concluded that doubling the amount of MA compared to the initiator brought about significant change in crystal structure through exertion of a steric effect (probably due to the grafting of MA), hence the predominant planar zigzag conformation.

#### After Biodegradation

All the buried films contained an extra peak compared with the nonburied samples of extruded PHB and chemically modified PHB. Generally seen at 2θ = 31.98°, the peak has been reported as indicative of soil particles (an XRD spectrum for soil is not given), confirming that some earth remained on the films despite the cleaning technique employed.

Iwata et al.^10^ states that the degradation of PHB fibers by a depolymerase isolated from *Ralstonia pickettii* T1 first leads to biodegradation of the planar zigzag conformation (β-form) and only subsequently to the 2/1 helix conformation (α-form), even though the β-form exists in the core region. Iwata et al.^10^ and Zhang et al.^62^ claim that the molecular chains of the β-form are more easily attacked by enzymatic molecules than α-form molecules, as steric hindrance to ester bonding in the planar zigzag conformation is lower than in the helix conformation. Therefore, it was expected that the degradation of β-form crystals would be emphasized in findings. The actual results were somewhat different, though, with β-form crystals recorded in all XRD spectra even after 90 days of exposure. In contrast, changes occurred at 2θ = 16.04° and 20.06° assigned to (020) and (110) planes (α-orthorhombic crystal structure), respectively. The authors consequently assumed that the α-orthorhombic crystal structure was destroyed simultaneously with β-form crystals, a phenomenon attributed to the immense diversity of soil microorganisms contributing variously toward PHB degradation.

XRD diffractograms of the extruded PHB and 0.5L contained a peak (020) that cleaved after 90 days of biodegradation. These results indicated a close correlation existed between the variations in the X-ray patterns in Figure 8 (before and after biodegradation) and changes in the dimensions of spherulites, as observed by SEM. Gazzano et al.^61^ state that differences in the ratio of intensity peaks (020) and (110) are instigated by the various degrees of orientation of the crystalline lamellae parallel to the film plane, in turn due to variation in spherulite size in films of limited thickness. They propose, with the aid of DSC analysis, that two distinct degrees of orientation of crystalline lamellae are present in the system. In the case of the extruded PHB herein, microbial degradation was the cause as soil microorganisms strongly impacted the thickness of the film and lamellae, as well as spherulite size during the biodegradation processes. In the case of the 0.5L specimen, however, this change was initiated by the aforementioned nucleation process.

### 3.6 Surface studies by fluorescence microscopy

Fluorescent staining was applied to the extruded PHB and chemically modified PHB films to visualize any attached biomass (live or dead bacterial cells), a technique which highlighted a gradual increase in microbial attachment to the polymer film surfaces over the time of exposure to them. Figure S6 shows the progress of colonization of the film surfaces by soil microorganisms, confirming three phases of biofilm formation: primo-colonization, growth, and stagnation. Figure S6 illustrates the slow increase in microbial adhesion for the extruded PHB film after 40, 60, and 90 days. In contrast, greater microbial attachment to the chemically modified PHB films was seen at the same intervals. The reason for this could have been variance in the hydrophilicity of the prepared films.^13^

**Figure 9:**
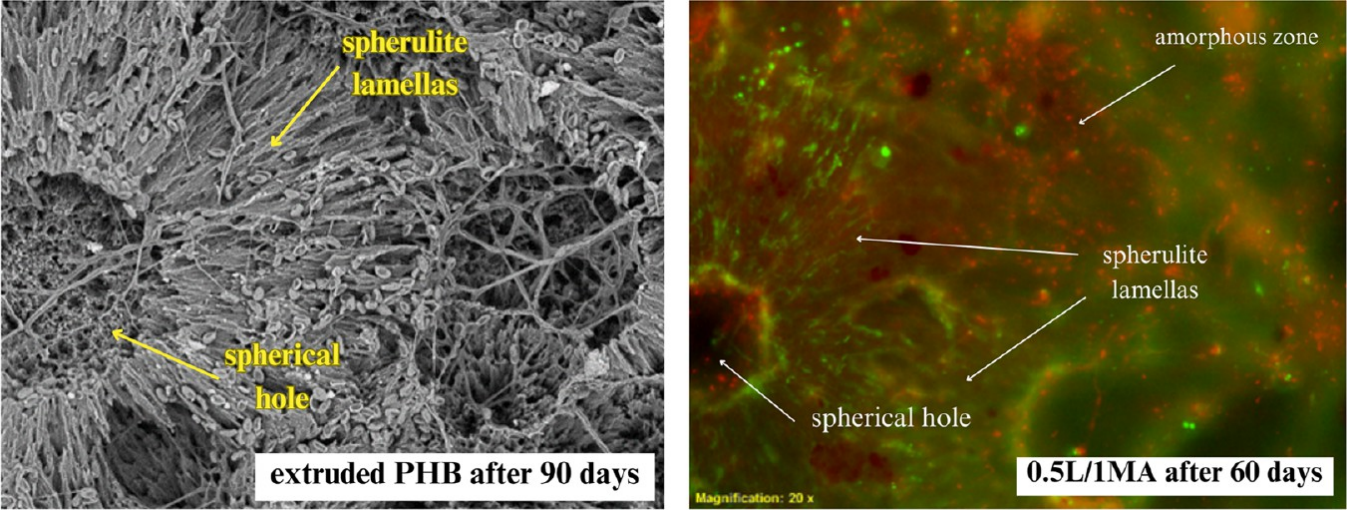
SEM image (left, 1000×) and fluorescence micrograph (right, 20×) of PHB-degrading microbial consortia in the process of degrading the polymer films (green, live; red, dead) after the biodegradation in the soil environment (55% soil humidity, 25 °C).

The fluorescence micrographs (Figure S6) indicated that, during decomposition of the amorphous phase on the surface, the microorganisms were evenly distributed over the entire surface of the film. Following biological decomposition of the amorphous phase, such metabolically active microorganisms transferred to the lamellar region of spherulites, accumulating in the interlamellar space (green). The comparison of SEM and fluorescence microscopy (Figure 9) was especially illustrative. The image shows virtually hollow central part of the spherulite where the amorphous polymer was probably the most accessible with the active cells (green) continuing their activity from the surface of the central cavity and microorganisms already in the stagnation phase in boundary areas between the spherulites (red). Fluorescence microscopy with the support of SEM thus corroborated the long held hypothesis that the amorphous phase is the most susceptible toward the microbial attack while the crystalline lamellae are the most recalcitrant in a very appealing way.

### 3.7 Sequencing Analysis of the Microbial Communities Present during Biodegradation

**Figure 10:**
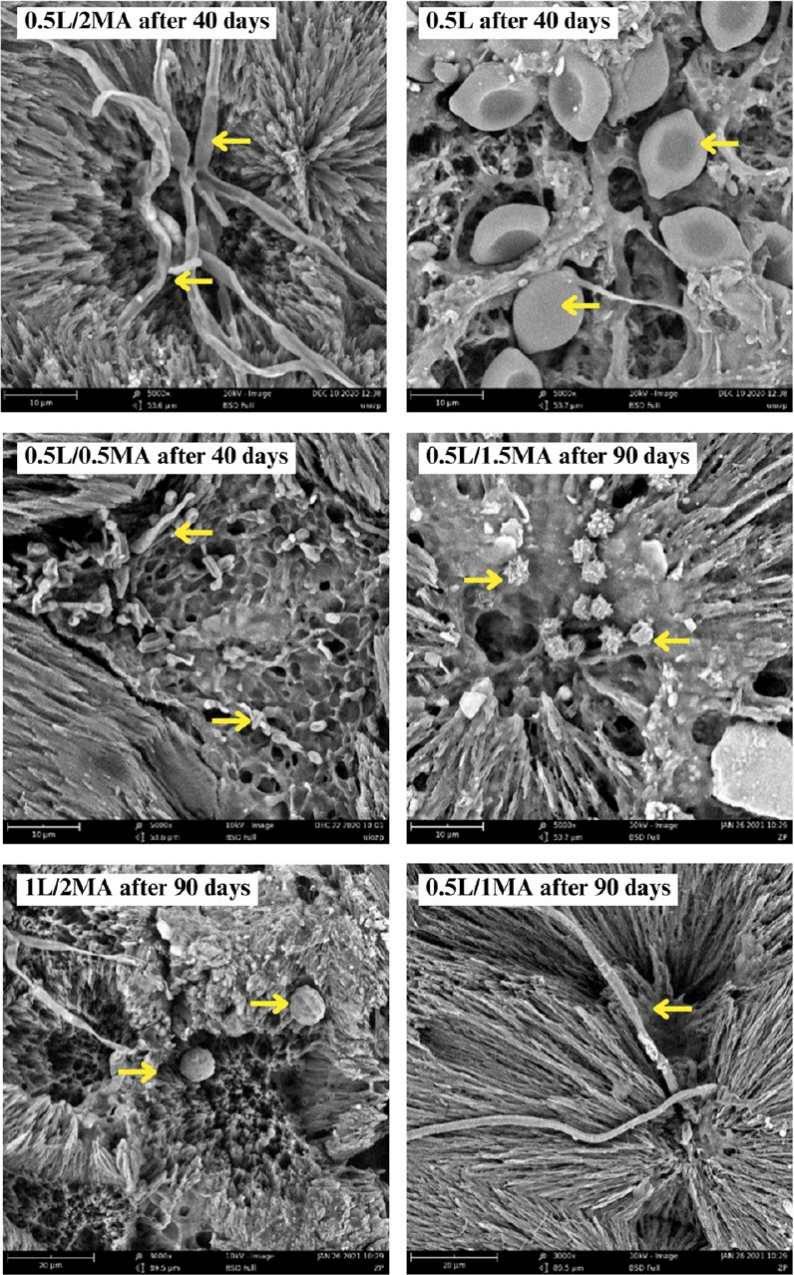
SEMs of spherulites and spherical cavities formed at their centers, as well as on boundary lines between spherulites during biodegradation in the soil environment by PHB-degrading microbial consortia: (a) fungal filaments, (b) fungal spores, (c) bacterial cells, (d) fungal spores, (e) fungal spores, and (f) fungal filaments.

A number of microorganisms that actively degrade PHB are described in the literature, including bacteria of various species and fungi.^63^ These microorganisms are not only limited to those equipped with the depolymerizing enzymes, but also species that utilize monomers and other PHB degradation products that occur in the environment due to the vital activity of primary PHB degraders. As detailed in the SEM image (Figure 10), spherical and rod-shaped cells are evident on the surfaces of the degraded films, and filaments of a greater thickness are also present, most likely microscopic fungi with their spores. Next-generation sequencing analysis was carried out to characterize the microbial communities on the surfaces of the films and to investigate the eventual differences between samples with different crystallinity and morphology.

**Figure 11:**
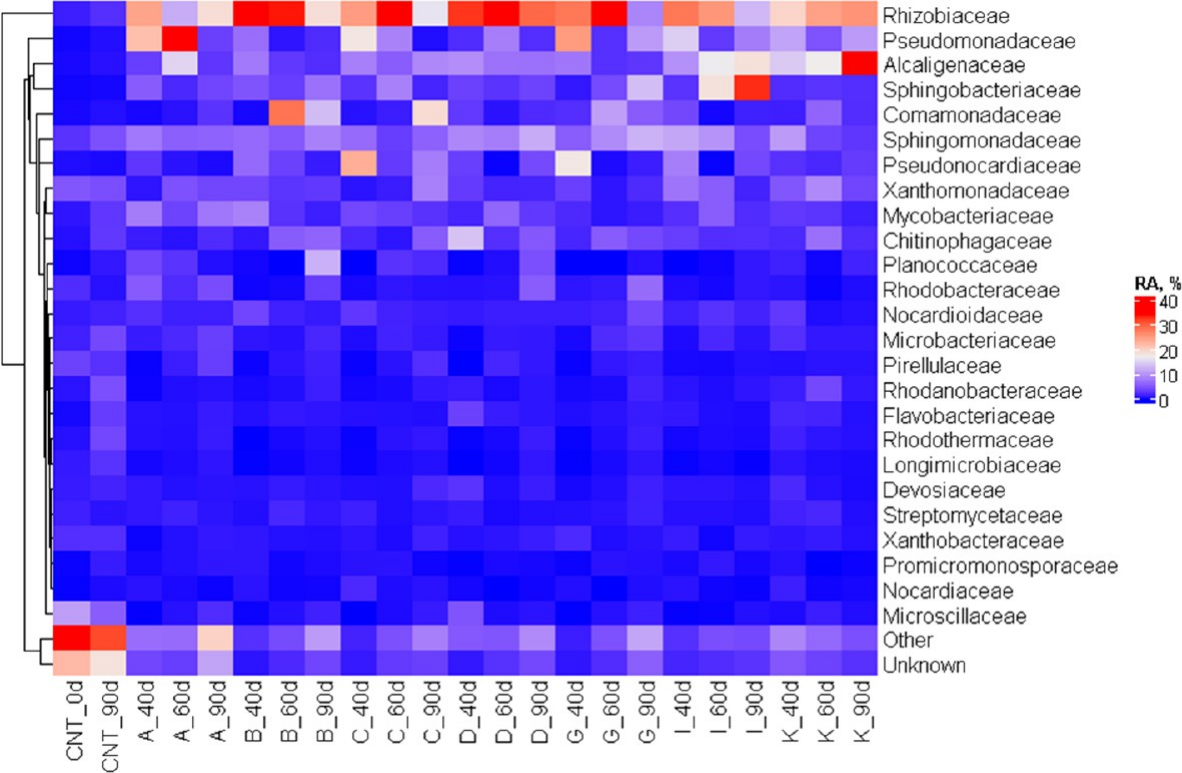
Bacterial community on the surfaces of the materials at 3 intervals, represented as a heatmap at a family taxonomic level (CNT, soil; A, extruded PHB; B, 0.5L; C, 0.5L/0.5MA; D, 0.5L/1MA; G, 1L; I, 1L/1MA; K, 1L/2L).

**Figure 12:**
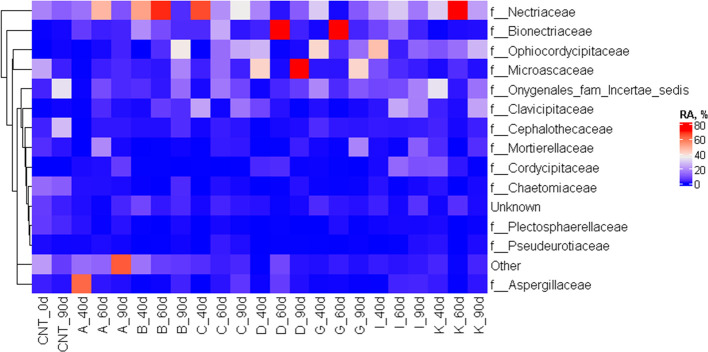
Fungal community on the surfaces of the materials at 3 intervals (40, 60, and 90 days), represented as a heatmap at family taxonomic level; (CNT, soil; A, extruded PHB; B, 0.5L; C, 0.5L/0.5MA; D, 0.5L/1MA; G, 1L; I, 1L/1MA; K, 1L/2L).

The composition of fungal and bacterial communities initially present in the soil and during the biodegradation on the surface of PHB and chemically modified PHB samples are presented in Figures 11 and 12.

The inherent community of soil bacteria was very diverse, with a majority belonging to the classes *Alphaproteobacteria*, *Gammaproteobacteria*, and *Actinobacteria*, which are typical, but without some more dominant bacterial families, the most intense signal pertains to a sum of minor taxa (Figure 11, “Other”). The situation was dramatically different on the surfaces of the investigated samples, where the diversity was lower (Figure S7). The community was dominated by several families, namely, *Rhisobiaceae*, *Pseudomonaceae*, *Alcaligenaceae*, and *Shingobacteriaceae,* which are all known to accumulate PHB and also have the capacity to utilize it.^64^ It seems that microbial diversity on most of the samples increased over time, as the PHB-utilizing community grew more complex (Figure S7). A clear difference was seen between the initial soil community and the PHB-degrading consortia (Figure S8), yet no apparent disparity existed between the various materials, indicating that the bacterial community was not significantly influenced by a variation in the crystallization patterns. This is not very surprising, because the opposite could mean that there are some enzyme systems and corresponding species that prefer materials with different morphologies. The result also suggests that the modified PHBs do not have ecotoxicological effects on the various members of the bacterial community and community structure as a whole.

According to the microscopic images, fungi participated very actively in the colonization of the materials. The majority of the fungi newly observed on the samples, in comparison with the control specimens, belonged to the *Hypocreales* order, but their family assignations were very diverse (Figure 12). Some of the families seen here as more active, namely, *Bionectriaceae* and *Nectriaceae*, where identified previously as PHB degraders,^65,66^ others often mentioned in the literature (*Aspergillaceae*^67^ and *Plectosphaerellaceae*(65)), where also present here but not highly abundant. Again, no apparent differences or pattern could be discerned between the sample type and fungi community composition. When comparing the background soil community with the ones on sample surfaces was obvious that *Bionectriaceae* and *Ophiocordycipitaceae* are dramatically more present on the samples and thus were suspected to be important for the biodegradation process.

## 4 Conclusions

To address environmental concerns arising from polyester degradation, an imperative lies in comprehensively investigating the interplay between chemical structure and morphology, specifically crystallinity, chain packing, and crystal surface, on the biodegradation of polyhydroxybutyrate (PHB) by natural microbial communities. Presented here is a short overview of novel findings:

- The extruded PHB and chemically modified PHB materials are characterized by a multistage (2–3) respirometric course of biodegradation that depends on the distribution of amorphous regions and crystal structure, respectively spherulite morphology. The first stage involves biodegradation of the readily available amorphous phase between the individual spherulitic units, followed by decomposition of the amorphous phase present in the interlamellar space of the individual spherulites. In the second and third stages, the crystalline phase is degraded, wherein morphology of the crystal structure (pertaining to the spherulites) exerts a significant impact on the rate and degree of biodegradation of crystalline zone.
- Microparticles of PHB films was detected and isolated, even though a high percentage of mineralization (70–80%) was determined.
- The nucleation of PHB crystals during the environmental processes retards the biodegradation of the PHB materials.
- According to the XRD analysis conducted, the orthorhombic α-form crystals with helical chain conformation are destroyed concurrently with β-form crystals of planar zigzag conformation.
- The crystal structure of PHB also influences the physiological behavior of the degrading soil microorganisms on the PHB surfaces.

This study focused on the biodegradation of PHB, and chemically modified PHB has augmented our comprehension of the behavior of PHB-degrading microorganisms contingent upon morphology and chemical structure. This newfound knowledge is instrumental for the molecular design of biodegradable polymers, yielding advantages in agricultural application wherein materials interface with soil throughout their service life. Such enhanced insights are poised to catalyze the development of environmentally friendly materials featuring a controlled lifetime.

## Supporting Information Available

FTIR spectra for the studied films; Macroscopic photographs of the extruded PHB and chemically modified PHB films before and after specific biodegradation periods in the soil environment; Biodegradation curves (soil burial tests at 55% soil humidity and 25 °C) for PHB in powder, extruded and chemically modified forms; Fluorescence micrographs of PHB-degrading microbial consortia in the process of degrading PHB; Diversity of the bacterial community on the surfaces of the materials at 3 intervals, described by Shannon and Simpson indexes; Scatter plot of principal component analysis, applying Nonmetric Multidimensional Scaling (NMDS) and Bray–Curtis dissimilarity to highlight resemblances between the bacterial communities on the investigated samples (PDF)
