# Supplementary material for: Comprehensive Biodegradation Analysis of Chemically Modified Poly(3-hydroxybutyrate) Materials with Different Crystal Structures
Source: Biomacromolecules. 2023 Oct 11;24(11):4939–57. doi: 10.1021/acs.biomac.3c00623 (PMC10646986; doi:10.1021/acs.biomac.3c00623)
Supplement: Supplementary file 1 — bm3c00623_si_001.pdf [file bm3c00623_si_001.pdf]

Supporting Information

Comprehensive biodegradation analysis of chemically modified poly(3-hydroxybutyrate) materials with different crystal structure

Markéta Julinová[†], Dagmar Šašinková[†], Antonín Minařík[‡], Martina Kaszonyiová[□], Alena Kalendová[□], Markéta Kadlečková[‡], Ahmad Fayyazbakhsh[†] and Marek Koutný[†]*

[†] Department of Environmental Protection Engineering, Faculty of Technology, Tomas Bata University in Zlín, Nad Ovčírnou 3685, 760 01, Zlín, Czech Republic

[‡] Department of Physics and Material Engineering, Faculty of Technology, Tomas Bata University in Zlín, Vavrečkova 5669, 760 01, Zlin, Czech Republic

[□] Department of Polymer Engineering, Faculty of Technology, Tomas Bata University in Zlín, Vavrečkova 5669, 760 01, Zlín, Czech Republic

*Corresponding author:

M. Julinová, julinova@utb.cz

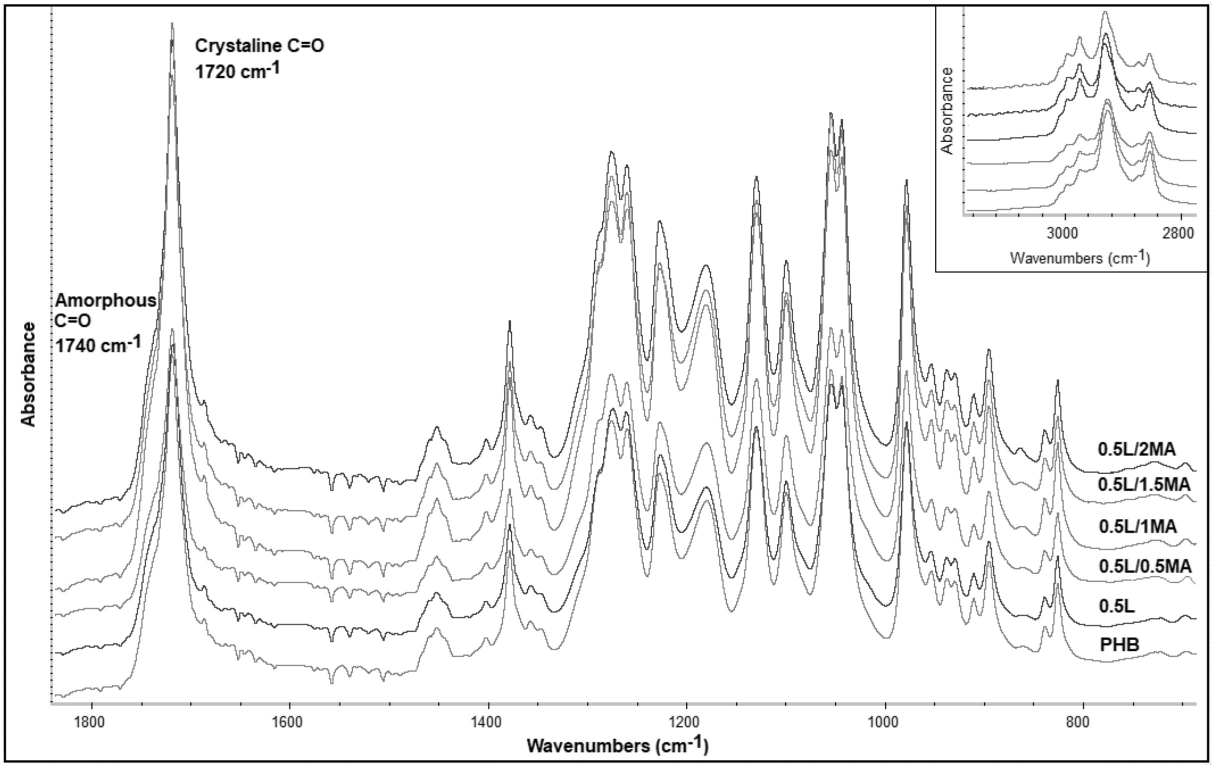

Figure S1 FTIR spectra for the studied films – 0.5% of the free-radical initiator.

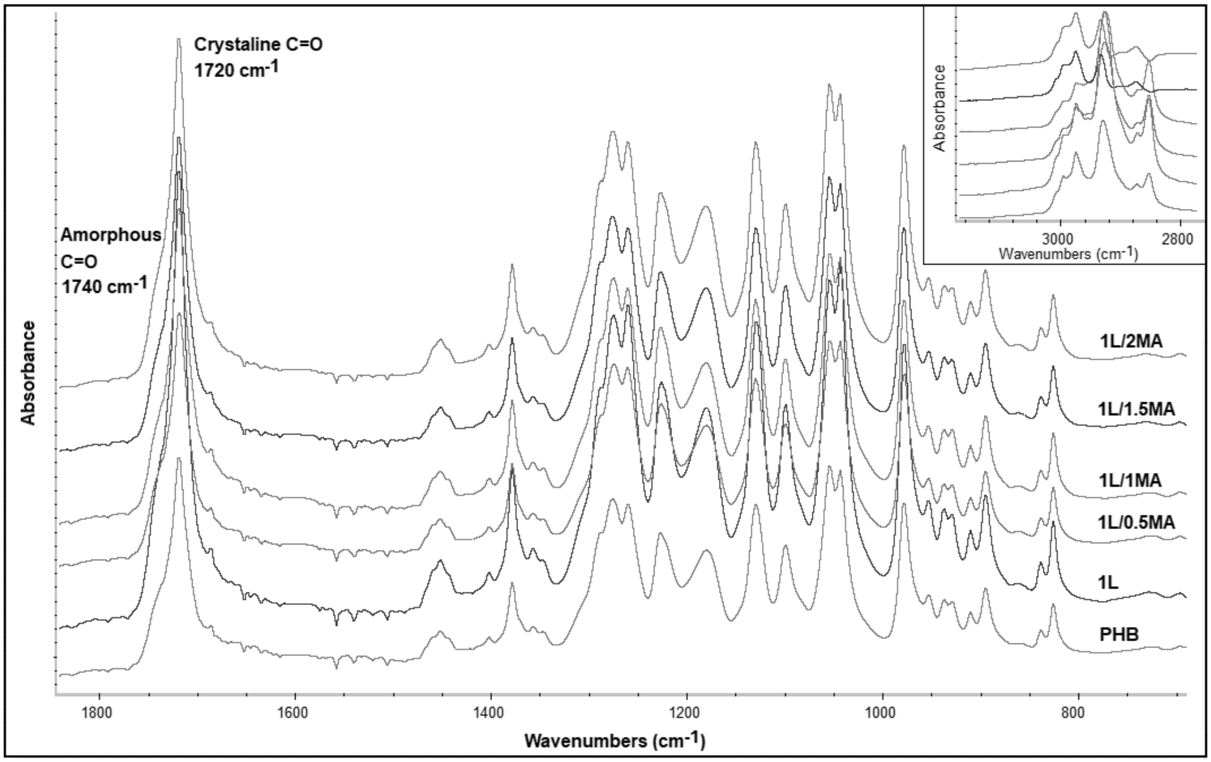

Figure S2 FTIR spectra for the studied films – 1% of the free-radical initiator.

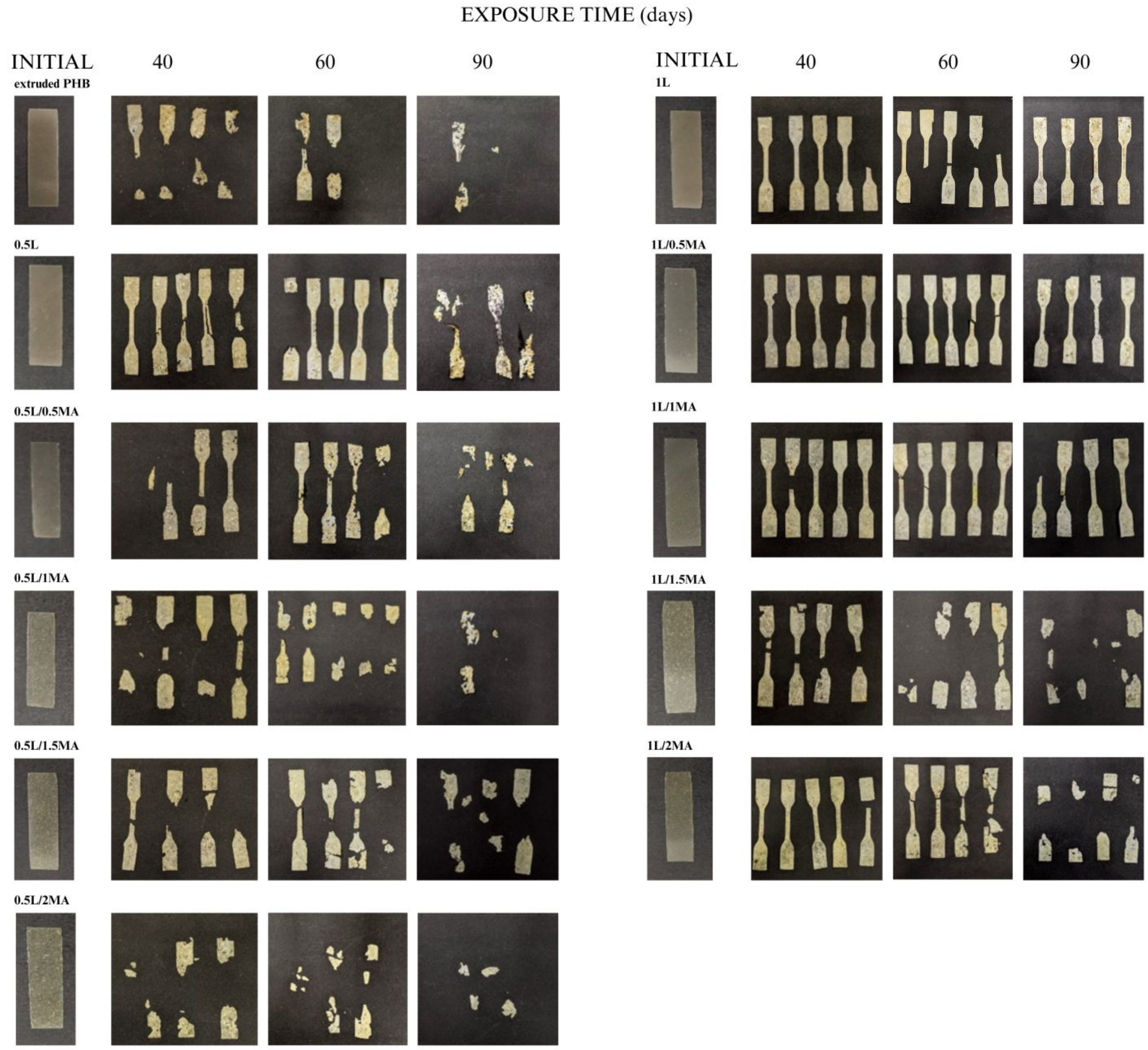

Figure S3. Macroscopic photographs of the extruded PHB and chemically modified PHB films before and after specific biodegradation periods in the soil environment (soil burial test, 55% soil humidity, 25°C).

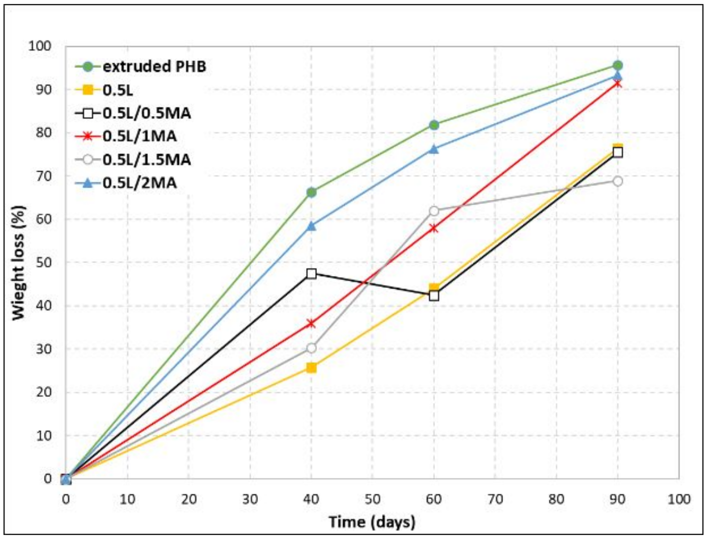

Figure S4. Soil burial tests at 55% soil humidity and 25°C for PHB in powder, extruded and chemically modified forms – 0.5% of the free-radical initiator.

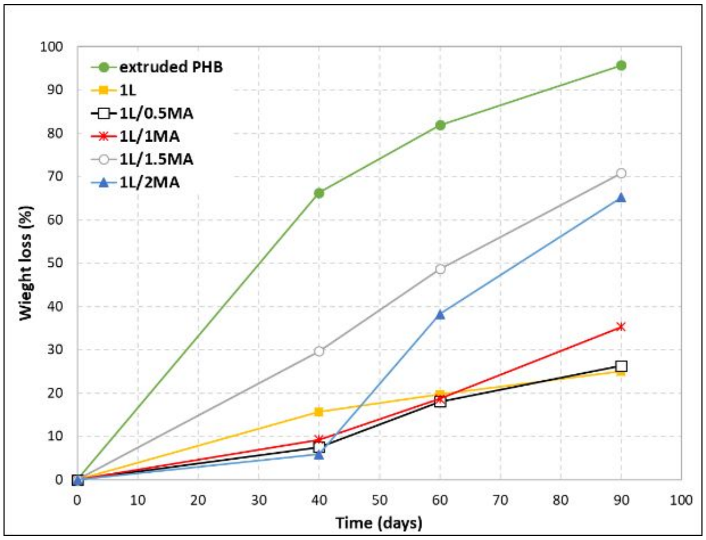

Figure S5. Soil burial tests at 55% soil humidity and 25°C for PHB in powder, extruded and chemically modified forms – 1% of the free-radical initiator.

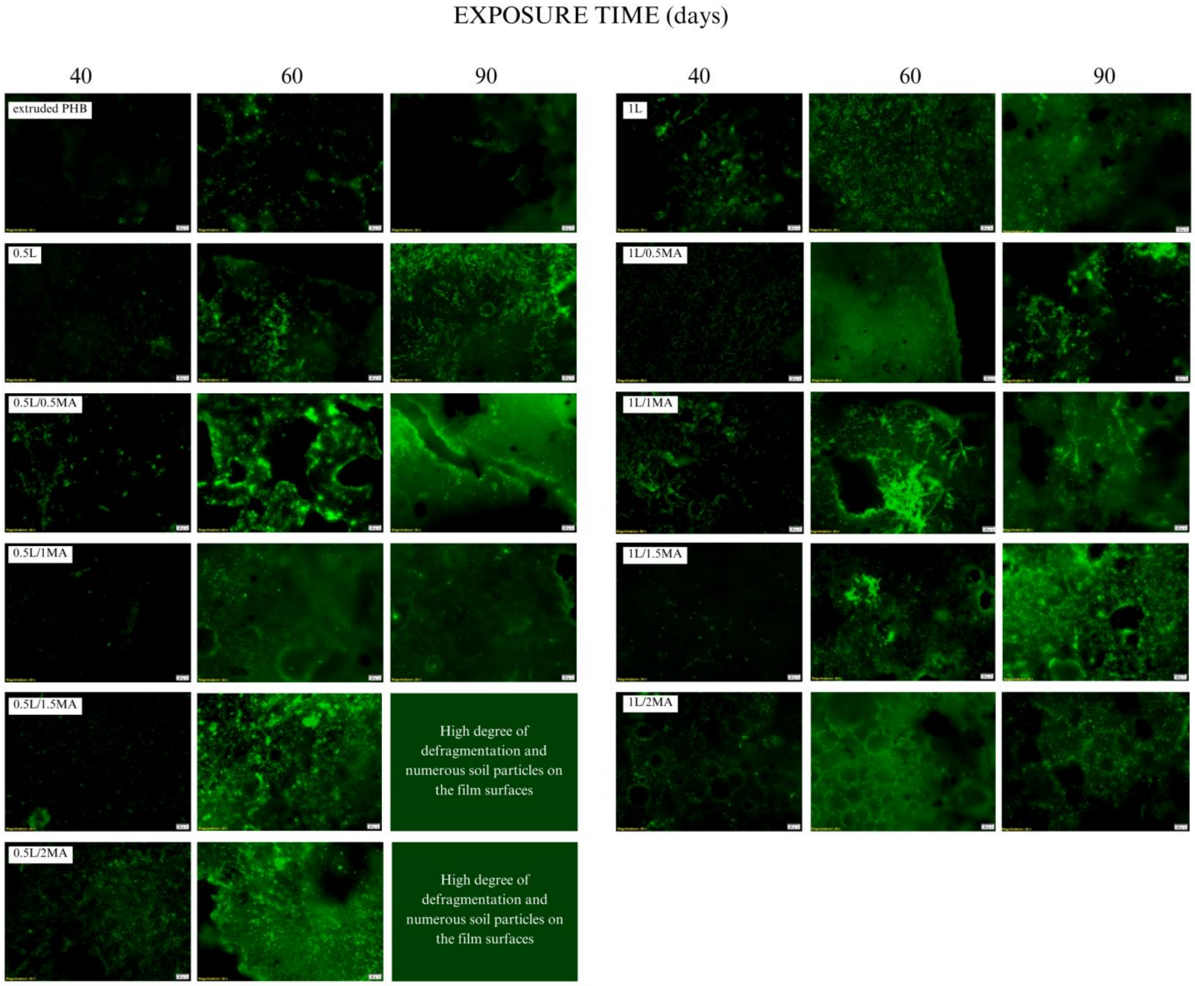

Figure S6. Fluorescence micrographs of PHB-degrading microbial consortia in the process of degrading PHB (green – live).

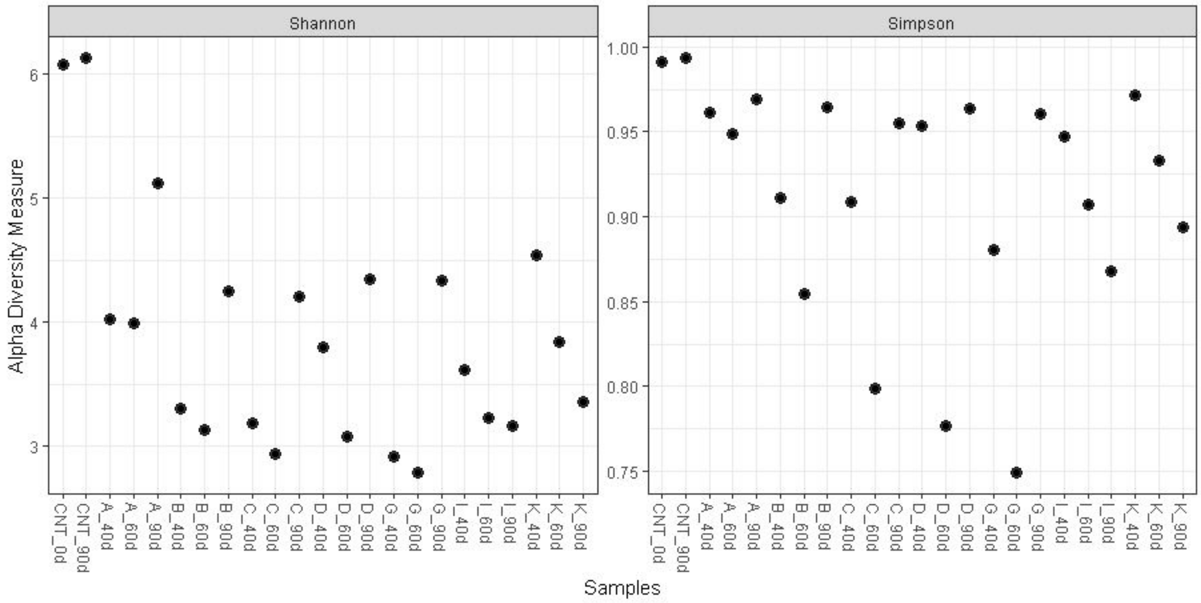

Figure S7. Diversity of the bacterial community on the surfaces of the materials at 3 intervals, described by Shannon and Simpson indexes; (CNT-soil, A – extruded PHB, B – 0.5L, C – 0.5L/0.5MA, D – 0.5L/1MA, G – 1L, I – 1L/1MA, K – 1L/2L).

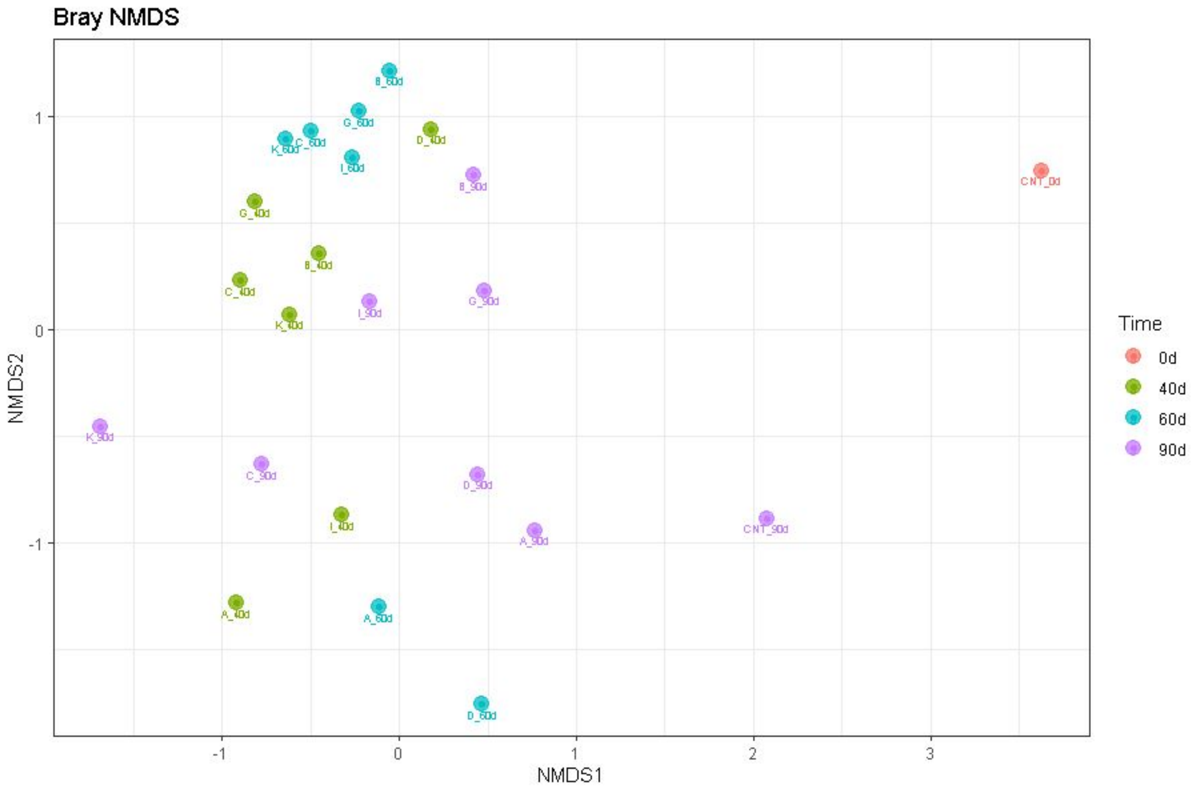

Figure S8. Scatter plot of principal component analysis, applying Non-metric Multidimensional Scaling (NMDS) and Bray-Curtis dissimilarity to highlight resemblances between the bacterial communities on the investigated samples; (CNT-soil, A – extruded PHB, B – 0.5L, C – 0.5L/0.5MA, D – 0.5L/1MA, G – 1L, I – 1L/1MA, K – 1L/2L).
